# Creation of Universal Primers Targeting Nonconserved, Horizontally Mobile Genes: Lessons and Considerations

**DOI:** 10.1128/AEM.02181-20

**Published:** 2021-01-29

**Authors:** Damon C. Brown, Raymond J. Turner

**Affiliations:** a Department of Biological Sciences, University of Calgary, Calgary, Alberta, Canada; University of Tartu

**Keywords:** PCR, genetics, mobile genetic elements, molecular methods, multidrug resistance, primer design, qPCR

## Abstract

Effective and accurate primer design is an increasingly important skill, as the use of PCR-based diagnostics in clinical and environmental settings is on the rise. While universal primer sets have been successfully designed for highly conserved core genes, such as 16S rRNA, and characteristic genes, such as *dsrAB* and *dnaJ*, primer sets for mobile, accessory genes such as multidrug resistance efflux pumps (MDREP) have not been explored. Here, we describe an approach to create universal primer sets for select MDREP genes chosen from five superfamilies (small multidrug resistance [SMR], major facilitator superfamily [MFS], multidrug and toxic [compound] extrusion [MATE], ATP-binding cassette [ABC], resistance-nodulation-cell division [RND], and proteobacterial antimicrobial compound efflux [PACE]) identified in a model community of six members (Acetobacterium woodii, Bacillus subtilis, Desulfovibrio vulgaris, Geoalkalibacter subterraneus, Pseudomonas putida, and Thauera aromatica). Using sequence alignments and *in silico* PCR analyses, a new approach for creating universal primer sets targeting mobile, nonconserved genes has been developed and compared to more traditional approaches used for highly conserved genes. The potential shortfalls of the primer sets designed this way are discussed. The approach described here can be adapted to any unique gene set and can aid in creating a wider, more robust library of primer sets to detect less-conserved genes and improve the field of PCR-based screening research.

**IMPORTANCE** Increasing use of molecular detection methods, specifically PCR and quantitative PCR (qPCR), requires utmost confidence in the results while minimizing false positives and false negatives due to poor primer designs. Frequently, these detection methods are focused on conserved core genes, which limits their applications. These screening methods are being used in various industries for specific genetic targets or key organisms, such as viral or infectious strains, or characteristic genes indicating the presence of key metabolic processes. The significance of this work is to improve primer design approaches to broaden the scope of detectable genes. The use of the techniques explored here will improve detection of nonconserved genes through unique primer design approaches. Additionally, the approaches here highlight additional, important information which can be gleaned during the *in silico* phase of primer design and will improve our gene annotations based on percent identities.

## Abstract (precis)

Increasing use of molecular detection methods, specifically PCR and quantitative PCR (qPCR), requires utmost confidence in the results while minimizing false positives and negatives due to poor primer designs. Frequently, these detection methods are focused on conserved core genes, which limits their applications.

## INTRODUCTION

PCR-based diagnostic approaches are being widely used for rapid screening of microbes and pathogens in environmental and clinical settings (1–4). Correct primer design is a critical factor in assessing the accuracy of the diagnostic approach to avoid false positives and false negatives. Many clinical studies focus on a single infectious strain or species (5–7) and thus the primers can be designed to focus on specific, characteristic genes present in the pathogenic strains and not in the benign strains, simplifying the primer design. However, this restricts the scope of use for the primers.

Some of the most successful attempts at designing “universal” primers are the 16S rRNA sets, of which there are many subsets, each targeting different variable regions of the 16S rRNA gene; they are reviewed elsewhere (8–10). These universal primer sets are mixed batches of primers with differing degrees of base variability at each position within the primer as denoted by the degenerate bases, where each possible combination is present and intended to target specific sequences, each representing a different species or groups of species. This approach attempts to cover the depth of diversity within the target location and provide unbiased detection of each species present before the first PCR cycle. Over the subsequent years, it has been shown that different primer sets have various detection levels for the species, both within different bacterial clades and between *Bacteria* and *Archaea* (8, 11, 12). Thus, the idea of “universal” is difficult even for an expectedly highly conserved core gene.

Alternatives to PCR-based primer detection are primer-probe assays such as fluorescence *in situ* hybridization (FISH), Southern blots, or microarrays, which all use primer binding to detect genes of interest but through different methodology approaches. While only PCR-based approaches are discussed here due to the ease of *in silico* analysis, it should be kept in mind that the approach to how primers are developed can be applied in these other techniques to answer other questions or for interpretation and conclusions from primer “hit” specificities.

Here, we describe an approach to design universal primer sets targeting multidrug resistance efflux pumps (MDREPs). MDREPs are nonconserved genes that encode a vast range of different proteins, from specific metabolite efflux transporters to those targeting specific groups of antiseptics and/or antibiotics (13–15). They are a group of integral membrane transport protein systems subdivided into six superfamilies: small multidrug resistance (SMR), major facilitator superfamily (MFS), multidrug and toxic (compound) extrusion (MATE), ATP-binding cassette (ABC), resistance-nodulation-cell division (RND), and proteobacterial antimicrobial compound efflux (PACE). A review of the superfamilies is available elsewhere (13). Due to the narrow range of annotated PACE genes (namely, *aceI*) and the absence of any annotated PACE genes present in the genomes of the model community members, the PACE genes were omitted from this study.

As their name suggests, MDREPs were originally classified based on their ability to confer resistance to antibiotics, although a single MDREP can have a diverse substrate range, sharing few structural, size, or ionic properties (16–18). To date, it is unclear how the specificity of the proteins is determined; however, recent research suggests it may be from different entrance channels allowing transport of chemicals with similar physicochemical properties or through the use of weaker hydrophobic interactions between substrate and efflux pump compared to the specific hydrogen bonds used by more-specific transporters (19, 20).

MDREPs are often found on mobile genetic elements, including plasmids, transposons, integrons, integrative conjugative elements, and genomic islands (15, 21–24). Thus, they are of particular and increasing importance due to their horizontal mobility and their contribution to the growing problem of global antibiotic resistance (24–26). The phenomenon of antibiotic resistance is well studied in medical environments but has only recently begun to be investigated in other environments, such as water treatment plants and activated sludges (27–29). Several research groups have begun using metagenomics to track and monitor the migration and abundance of different MDREP genes in wastewater following various treatment methods, with mixed results (30, 31). Our interest is to follow specific MDREPs in the context of biocide resistance in a model community of six members designed to resemble a microbiologically influenced corrosion environment.

Many detailed reviews of the different efflux pump superfamilies have been published (32–37); they have been used for the selection of targets in this study and are shown in Table 1. Here, gene targets have been specifically selected for their published substrate compounds, focusing on antiseptics/biocides. While there are some conserved regions and motifs within the MDREP superfamilies, these traits are typically limited to very short regions, as in the conserved N terminus region of AcrB (38), specific residues, as in the proton relay components of transmembrane helices (39), or short motifs, as in the case of conserved residues in the motif C of members of the MFS superfamily (40). There are no known residues or motifs conserved across all superfamilies and those within a superfamily are less conserved than one would assume (41).

**TABLE 1:** Multidrug resistance efflux pump genes targeted in this study and their details

Superfamily	Gene	Substrates	Reference (s)
MFS	*emrB*	Carbonyl cyanide m-chlorophenylhydrazone (CCCP), tetrarchlorosalicyl anilide, nalidixic acid, thioloactomycin	44, 45
	*qacA*	Ethidium bromide, cetrimide, benzalkonium chloride, chlorhexidine	46
MATE	*norM*	Norfloxacin, doxorubicin, acriflavine	47
	*mepA*	Tigecycline, pentamidine, ethidium bromide, dequalinium, tetraphenylphosphonium, 1-(4-trimethylammoniumphenyl)-6-phenyl-1,3,5-hexatriene *p*-toluenesulfonate, chlorhexidine, norfloxacin, ciprofloxacin	48, 49
RND	*acrB*	Taurocholate, glycocholate, tetracycline, chloramphenicol, fluoroquinolone, novobiocin, erythromycin, fusidic acid, rifampin, ethidium bromide, acriflavine, crystal violet, sodium dodecyl sulfate, deoxycholate, nafcillin, cloxacillin,	50–52
	*mexB*	Quinolones, macrolides, tetracyclines, lincomycin, chloramphenicol, novobiocin, oxacillin, ethidium bromide, tetraphenylphosphonium, sodium dodecyl sulfate	53, 54
SMR	*ebrAB*	Ethidium bromide, tetraphenylphosphonium chloride, acriflavine, pyronine Y, safranin O	55, 56
	*emrE*	Quaternary cation/ammonium compounds (e.g., betaine, choline, methyl viologen, tetraphenylphosphonium, benzalkonium, cetyltrimethylammonium bromide, cetylpyridinium chloride), ethidium bromide, acriflavine, crystal violet, pyronine Y, safranin O	57, 58
	*qacE*	Ethidium bromide, proflavine, rhodamine, cetyltrimethylammonium bromide, benzalkonium chloride, tetraphenylarsonium chloride, diamidinodiphenylamine dichloride, propamidine isethionate, pentamidine isethionate	46, 59
	*sugE*	Tributyltin, ethidium bromide, chloramphenicol, tetracycline, cetylpyridinium, cetyldimethylethyl ammonium, cetrimide	60, 61
ABC	*lmrA*	Vinblastine, vincristine, daunomycin, doxorubicin, colchicine, ethidium bromide, valinomycin, nigericin, Hoechst 33342, diphenylhexatriene, rhodamine 6G	62

We explore the possibility of designing universal primers for nonconserved, mobile gene targets in the fashion of the multiple universal 16S rRNA primer sets (2, 8). We will discuss difficulties and challenges encountered and describe a novel approach which can be applied to any desired gene target for improved detection. This novel primer design approach was tested on environmental water samples treated with various biocides.

## RESULTS AND DISCUSSION

**FIG 1:**
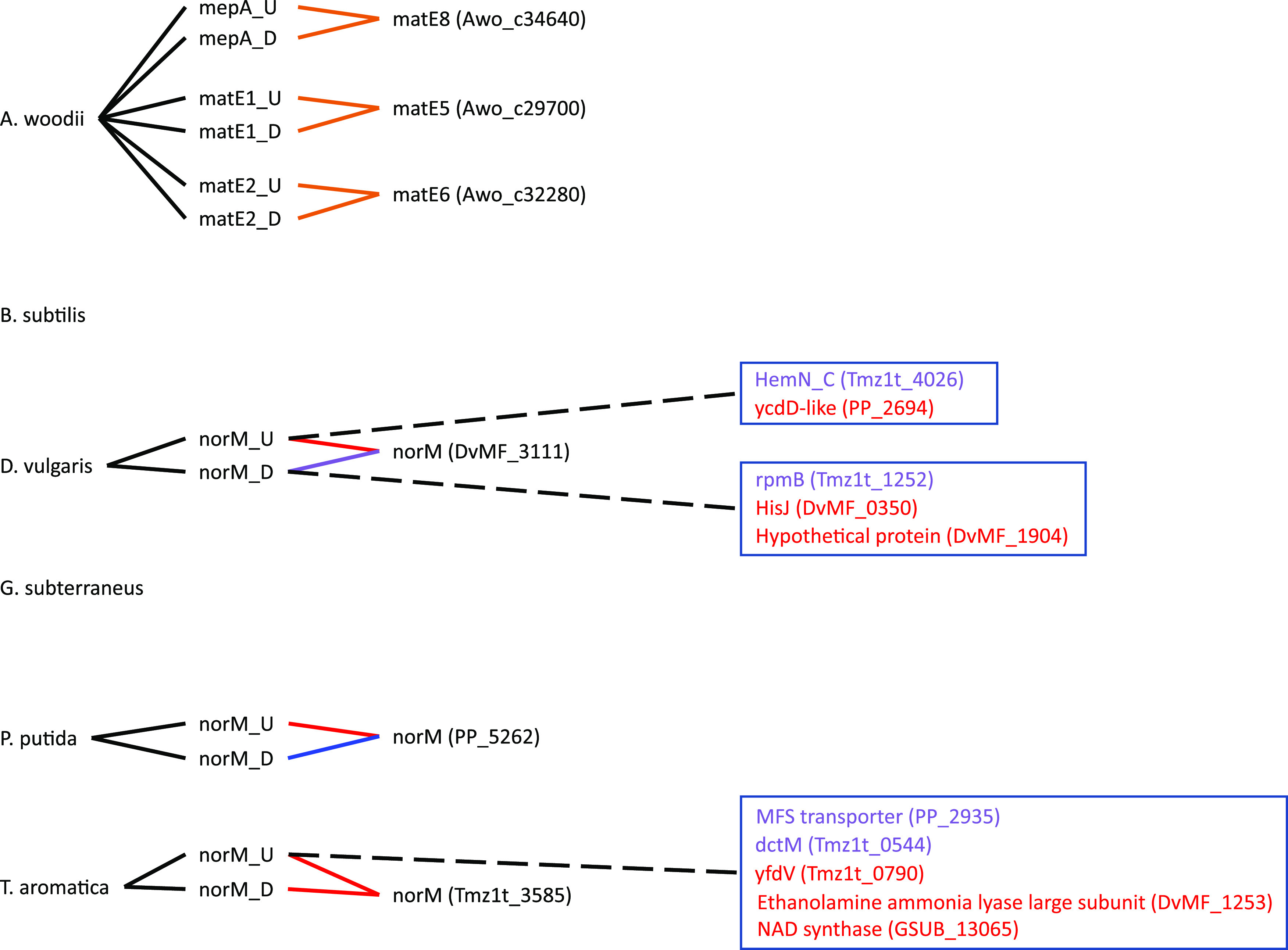
Visual representation of the primers designed in this study targeting the multidrug and toxic (compound) extrusion (MATE) genes and all targets to which they bind. The type of line connecting the primer and the target indicates the percent identity of binding (solid line 100%, dashed line 90.0 to 99.9%, dotted line 80.0 to 89.9%). Color coding is used to indicate the melting temperature range divided into 5°C increments (light green ≤49.9°C, orange 50.0 to 54.9°C, blue 55.0 to 59.9°C, purple 60.0 to 64.9°C, red 65.0 to 69.9°C, and dark red ≥70.0°C). A black dashed line connecting to the gene targets grouped into a box indicates the percent identity of all genes in the box and the text color represents the melting temperature.

The goal of this study was to go through a primer design workflow, then test the efficacy of the designed primer sets *in silico* to evaluate the success or failure of the design. The approach is to evaluate the primers before using them experimentally, consuming resources and time in optimization. The output of our *in silico* primer annealing experiment is represented over four figures that illustrate all the binding locations for each of the primers within the model community genomes, separated into MDREP superfamilies (Fig. 1 to Fig. 4). The results illustrate the direct hits toward the intended MDREP gene but also the unintended binding locations where primers would anneal under different annealing temperatures and with different sequence homologies (see Table 2 for primer details). In all figures, the type of line used indicates the percent identity of the sequence (solid line 100%, dashed line 90.0 to 99.9%, dotted line 80.0 to 89.9%) and the color coding is used to indicate the melting temperature range divided into 5°C increments (light green ≤49.9°C, orange 50.0 to 54.9°C, blue 55.0 to 59.9°C, purple 60.0 to 64.9°C, red 65.0 to 69.9°C, and dark red ≥70.0°C). For simplicity, the term “hypothetical protein” refers to all genes annotated as encoding a “conserved protein of unknown function,” “hypothetical protein,” “conserved hypothetical protein,” “conserved protein of unknown function,” or “conserved exported protein of unknown function.”

**FIG 2:**
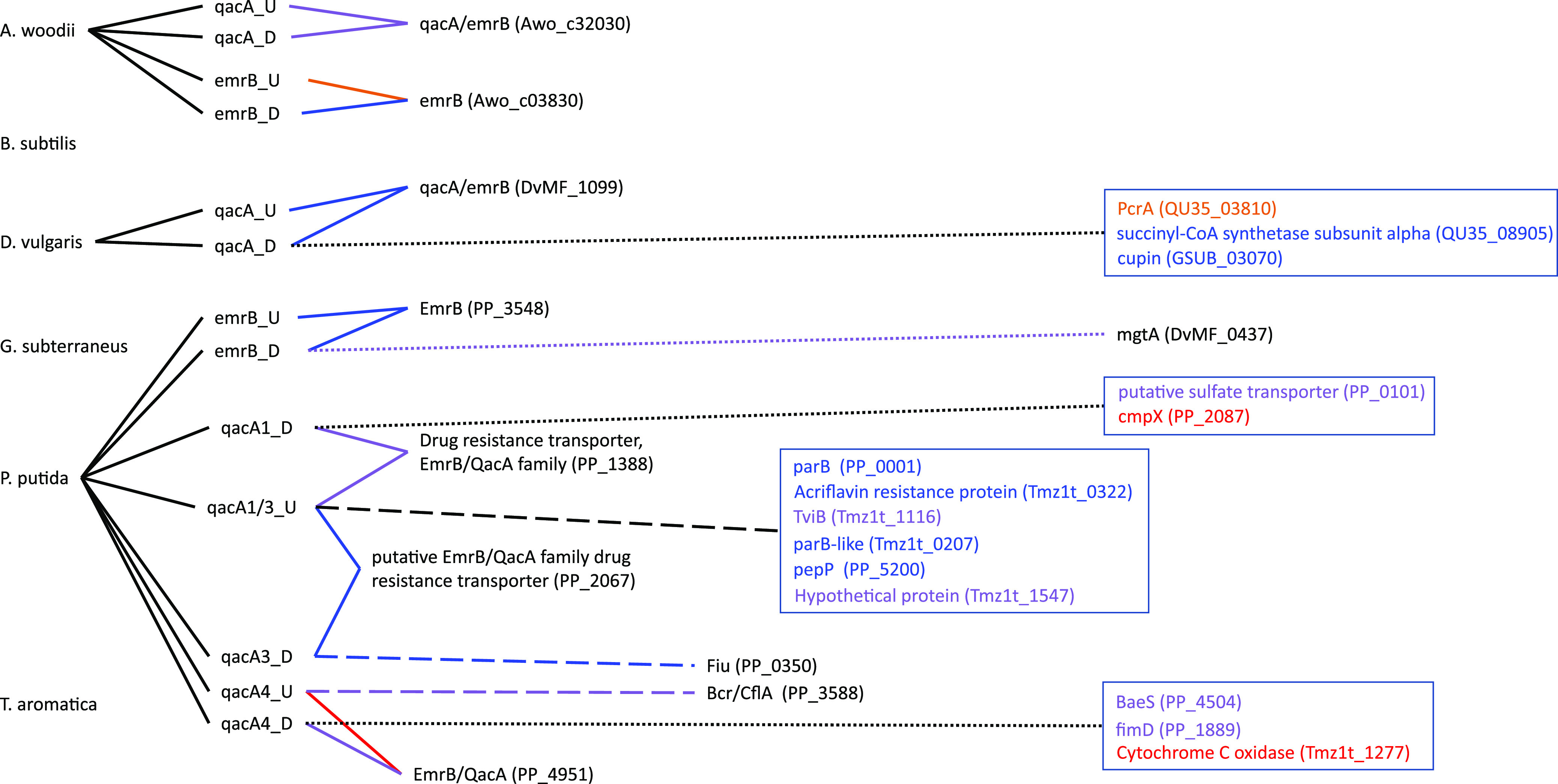
Visual representation of the primers designed in this study targeting the major facilitator superfamily (MFS) genes and all targets to which they bind. The type of line connecting the primer and the target indicates the percent identity of binding (solid line 100%, dashed line 90.0 to 99.9%, dotted line 80.0 to 89.9%). Color coding is used to indicate the melting temperature range divided into 5°C increments (light green ≤49.9°C, orange 50.0 to 54.9°C, blue 55.0 to 59.9°C, purple 60.0 to 64.9°C, red 65.0 to 69.9°C, and dark red ≥70.0°C). A black line connecting to the gene targets grouped in a box indicates the percent identity of all genes in the box and the text color represents the melting temperature.

**FIG 3:**
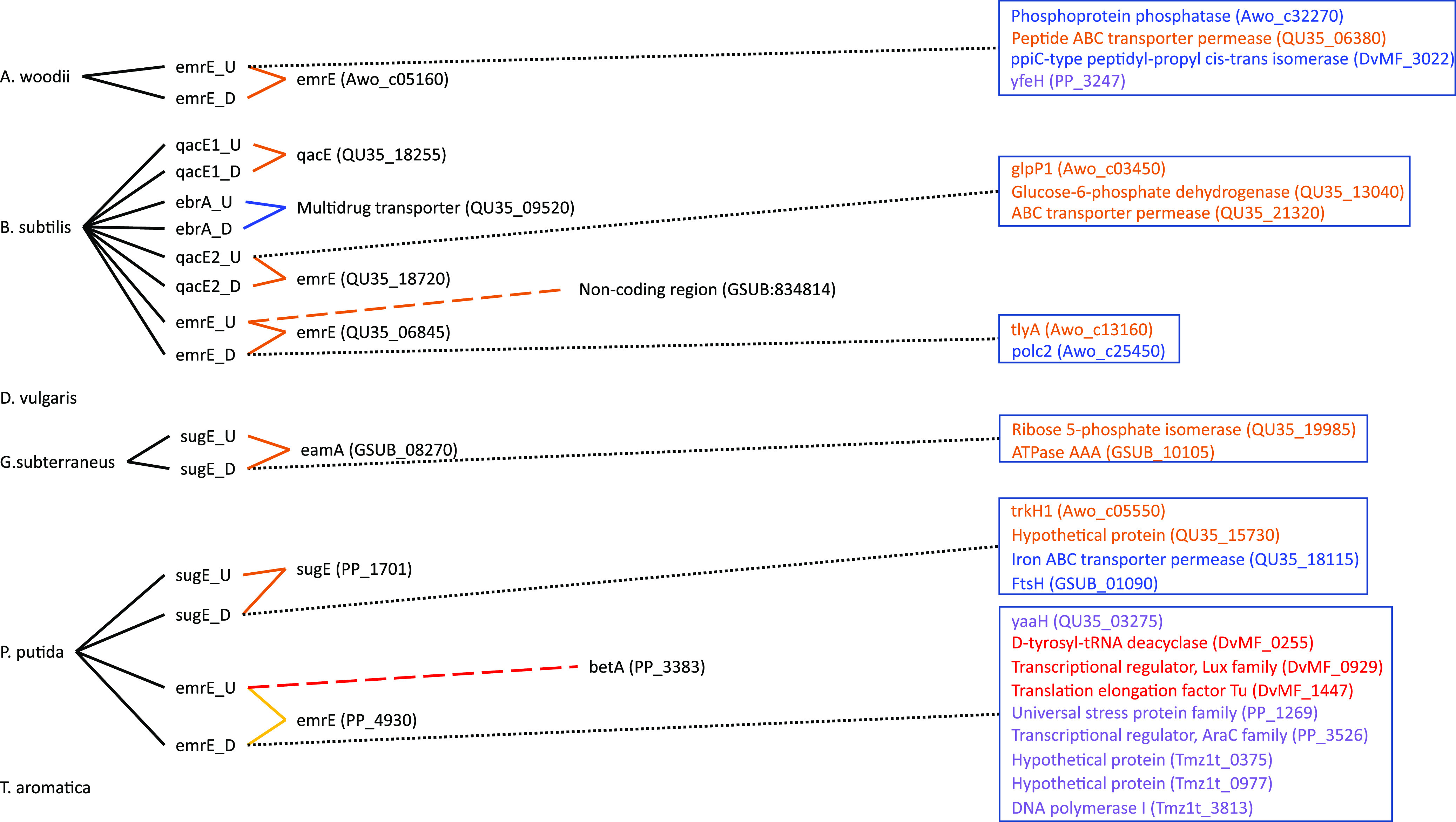
Visual representation of the primers designed in this study targeting the small multidrug resistance (SMR) genes and all targets to which they bind. The type of line connecting the primer and the target indicates the percent identity of binding (solid line 100%, dashed line 90.0 to 99.9%, dotted line 80.0 to 89.9%). The color coding is used to indicate the melting temperature range divided into 5°C increments (light green ≤49.9°C, orange 50.0 to 54.9°C, blue 55.0 to 59.9°C, purple 60.0 to 64.9°C, red 65.0 to 69.9°C, and dark red ≥70.0°C). A black line connecting to the gene targets grouped into a box indicates the percent identity of all genes in the box and the text color represents the melting temperature.

**FIG 4:**
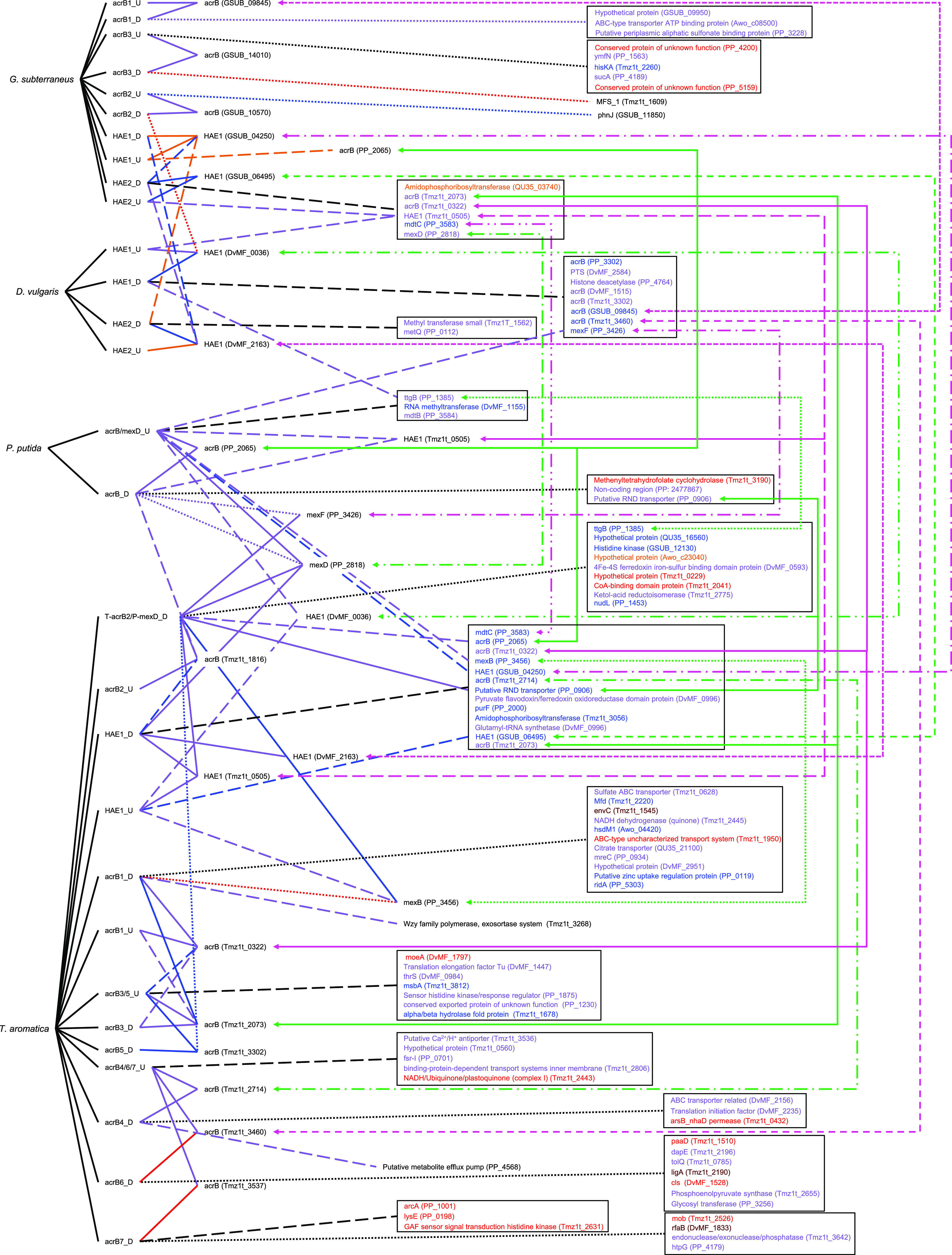
Visual representation of the primers designed in this study targeting the resistance-nodulation-cell division (RND) genes and all targets to which they bind. The type of line connecting the primer and the target indicates the percent identity of binding (solid line 100%, dashed line 90.0 to 99.9%, dotted line 80.0 to 89.9%). The color coding is used to indicate the melting temperature range divided into 5°C increments (light green ≤49.9°C, orange 50.0 to 54.9°C, blue 55.0 to 59.9°C, purple 60.0 to 64.9°C, red 65.0 to 69.9°C, and dark red ≥70.0°C). A black line connecting to the gene targets grouped into a box indicates the percent identity of all genes in the box and the text color represents the melting temperature. Green and pink lines on the right link gene targets which have been duplicated to simplify visual representation.

**TABLE 2:** PCR primer details and amplification protocols

Species	Targeted gene(s)^*a*^	Sequence (5′–3′)^*b*^	Annealing temp (°C)	GC content (%)
Acetobacterium woodii	*emrB*	F- CCT GTT TAC ATT GGG GTC GTT	54.8	48
		R- AAC CAA AGA TCC CAA GGC GA	55.9	50
	*emrE*	F- TTG CCT TGG GAA TCA TGA TTC T	54.6	41
		R- GTG ATG TTC GTG GAC TGT TTT C	54.4	45
	*matE1*	F- CTG GCT GCC CTG ACT AAT AA	53.4	50
		R- GCG TTT CCG ATC CAA ATG AT	53.3	45
	*matE2*	F- AAA TTG GAA TCA ACC AGG CG	53.4	45
		R- CGT CAA TGT TTG GAG TAG CC	53.3	50
	*mepA*	F- CAT TAC TCT TTG GTG TCG GC	53.3	50
		R- GCG GTA TAA GGG ATC GCA TA	53.5	50
	*qacA*	F- GCC GCC CCA ACG AGT CCT TT	61.9	65
		R- GGG ATG GGC GCC GGA ATG TT	62.0	65
Bacillus subtilis	*ebrA*	F- TAC TCC GAT CAC TGT CGT CAG C	58.0	55
		R- CCT CAC GAT TGC CAT ATG TTC GG	58.0	52
	*emrE*	F- TTG ACT GTG CTT TCT TTT TCG G	54.2	41
		R- TGG TAA GAG ACA ATC CGC AAA A	54.4	41
	*lmrA*	F- GAT AAA ATT CAC GCG GCA GT	53.7	45
		R- CCG GGC GGT ATT TTA AAT GG	53.7	50
	*qacE1*	F- CAG GTT TAA AGA CAC AAC ACC G	54.3	45
		R- ACA AAG CTT ATT CCC AGT TTG C	53.9	41
	*qacE2*	F- ATT ATA GCT GCC ATT GCC ATG A	54.3	41
		R- GTG AAA TCA CCT GTG AAA GCT G	54.3	45
Desulfovibrio vulgaris	*HAE1*	F- CCC TAC GAC ACC ACC CGC TT	60.7	65
		R- GAT GAT GGC GTC GTC CAC CA	59.0	60
	*HAE2*	F- AAT GTG TTG ATG GAG AAG CCC	54.8	48
		R- ATC CCC TAC GAC ACC ACC AA	56.8	55
	*norM*	F- ACG GCC TGC CCA GCG GCA TC	66.8	75
		R- GCT GCC CTT GCC CAT GGC CT	64.4	70
	*qacA/emrB*	F- AGA AGA TCC ACC GCC AGG TG	58.3	60
		R- TCC TGT TCC GCA TTT TTC AGG	55.4	48
Geoalkalibacter subterraneus	*acrB2*	F- TGA AGT CCT GCC GCC AGT CAT	60.9	59
		R- GCG TAA AAA GTC ACC GGC ACC A	60.3	55
	*acrB1*	F- AGG AAC GCC TTT TGG ATG ACG C	60.1	55
		R- CCC TGG CAG GTC AGA CCA AGA A	60.2	59
	*acrB3*	F- GCC GCA TGA ACC TGC TGA TCA A	60.2	55
		R- CAC ACC CAG CGC CAT GAT GAA G	60.5	59
	*sugE*	F- TAG CCG GAT TAT TTG AAG TCG	52.2	43
		R- CCG AAA AGA ATA ATC CCG AGA A	52.4	41
	*HAE1*	F- ACA TTT TCC ACC ACC ACA AT	52.1	40
		R- ATT CCC TAC GAT ACC ACC AA	52.0	45
	*HAE2*	F- CCC TAT GAC ACC ACG CCT TT	56.4	55
		R- CAC GAT GGC GTC ATC CAC CA	59.3	60
Pseudomonas putida	*emrB*	F- AGA AGA TCC ATG GCC AGC TG	56.2	55
		R- TGG GCT TTC GTG TCT TGC AGG	59.7	57
	*emrB/qacA*	F- GAT CAC CTC GCC AAT CTG CA	56.9	55
		R- CTG GTC AGC CTG ATC ACC TT	55.7	55
	*norM*	F- TGG GCC TGC CGA TTG GCG GT	65.9	70
		R- GTT GCC AGC GCC GTA GTA CA	59.6	60
	*mexB*	F- AAA TCG GTG CCC AGG AAT ACC A	58.1	50
		R- TGT TGA TGA CCT GCT CGA TCG A	57.9	50
	*sugE*	F- GAC TCG CCG AAC AGA ATG AT	54.6	50
		R- TGT CCT GGA TCA TCC TGT TTT T	53.7	41
	*qacA1/3*	F- AGA A**S**A **Y**CC AGC GCC ACG A**M**	58.3–61.8	55–65
	*qacA1*	R- TGC TGG CCC GTG TAC TGC AGG	63.5	67
	*qacA3*	R- TCG TAA TCC GGG TGA TCC AGG	57.6	57
	*qacA4*	F- CGC GTG GTG CAG GGC CTG GG	67.3	80
		R- CCA AGC AGG CCG ACT GGC AGG	64.4	71
	*acrB/mexD*	F- TGG **Y**GG CGC **W**GT ACG AAA GC	60.1–62.8	60-65
	*acrB*	R- TTG GCG AAC GCC ACC ATC AGG AT	63.5	57
Shared	*Taro_acrB2/Pputi_mexD*	R- TTG GCG AAC TC**S** A**Y**G ATC AGG AT	58.1–60.2	48-52
Thauera aromatica	*acrB1*	F- CTA CAT CGT CGT ACC GTG GGC A	60.5	59
		R- ATC AGC GAG ACC GTC ATC AGC A	60.2	55
	*acrB2*	F- TGG CAG CGC AGT TCG AGA GC	62.2	65
	*acrB4/acrB6/acrB7*	F- ACC A**R**C A**W**G CCG AGC GCG AT	61.9–64.1	60–65
	*acrB4*	R- GGG CAT GGA GCT GAA CGT GGT	62.5	62
	*acrB6*	R- CGG CAT CCG CCT CGA GCG CGT	69.5	76
	*acrB7*	R- GGG CGT GCA GCT GCG CCT GAT	68.0	71
	*acrB3/acrB5*	F- AGC GCG AT**S** ATG CCG A**Y**C AT	60.5–61.5	55–60
	*acrB3*	R- AGT TGC TGT GGG GCG GCG AG	64.3	70
	*acrB5*	R- AGT TGA AGT GGG ACG GCG AG	59.8	60
	*norM*	F- TCG GCC TGC CGA TGG GGG TG	65.6	75
		R- GTC CTG CGC GCC GGC CGA CT	69.0	80
	*HAE1*	F- CCC TAC GAC ACC ACG CCC TT	60.7	65
		R- CAC GAT GGC GTC GTC CAC CA	61.6	65

a Gene numbers are arbitrary, according to their annotation order as they appeared in the respective genome.

b F-, upstream primer; R-, downstream primer; degenerate (boldface) base codes: Y = C, T; W = A, T; R = A, G; S = G, C; M = A, C.

This work illustrates the difficulty of making a universal primer set for accessory/character genes compared to core genes. The challenge is further highlighted working with genes that frequent mobile genetic elements, thus providing the opportunity for increased divergence. To illustrate the issues we observed and our findings, we discuss a few key examples highlighting certain trends and difficulties which were discovered as a result of this primer design work.

### Off-target unintended primer binding.

To begin, we discuss an example showing the potential for a primer designed for one target to identify other MDREPs in another species. The upstream *norM* primer targeting Thauera aromatica has a relatively high annealing temperature on its target sequence (65.6°C), owing to its high GC content of 75%. This primer has five unintended binding targets, all with a percent identity of 90.0 to 99.9% (Fig. 1). Two of these sites are located within T. aromatica and target *dctM* (Tmz1t_0544) and *yfdV* (Tmz1t_0790). *dctM* encodes a tripartite ATP-independent periplasmic transporter which falls under the C_4_-dicarboxylate transport system classification according to KEGG, while *yfdV* encodes an auxin efflux carrier that is only a hypothetical protein with general function predicted and thus could be a more general transporter. The other unintended targets include genes encoding a conserved membrane protein of unknown function in Pseudomonas putida (PP_2935) that is provisionally in the MFS superfamily, an ethanolamine ammonia lyase large subunit (DvMF_1253), and an NAD synthase (GSUB_13065). The annealing temperatures of these locations are at or just below the intended annealing temperature. This illustrates a clear example of a primer targeting a sequence of nucleotides in what may be a conserved region of certain transporter genes. It is important to note that although these primers are detecting these unintended targets *in silico*, in each case only a single primer is binding and therefore no double-stranded PCR product would be formed *in vitro*. In practice, these unintended single primer interactions will only affect PCR amplification by reducing the availability of the primers (i.e., primer efficacy) for finding the intended sequence. Significant amounts of off-target binding interactions will reduce the availability of the primers to bind to the intended target sequence, which decreases the amount of PCR amplicon production. This reduction in primer availability has additional implications for interpreting qPCR data, as it may result in an underestimation of gene copies. This issue may also be addressed in wet lab work through an iterative screening process to determine the ideal primer concentration range for each primer to account for unintended binding. However, using the approach here should cut down on such labor. A consideration is that this ratio may vary depending on the genomic template being used, e.g., pure culture DNA compared to complex environments.

A more complicated example of a primer with unintended binding interactions is the downstream *qacA* primer for *Desulfovibrio vulgaris* (Fig. 2). In this example, the downstream *qacA* primer has an annealing temperature of 55.4°C and three unintended binding sites, none of which are in the *D. vulgaris* genome. The primer binds at the same or a lower annealing temperature to *pcrA* (QU35_03810), encoding an ATP-dependent DNA helicase, and a gene for the succinyl-CoA synthetase alpha subunit (QU35_08905), both in Bacillus subtilis, as well as to a cupin gene (GSUB_03070) in *Geoalkalibacter subterraneus*. This example illustrates undesired targeting that does not provide any sort of additional information, such as potentially conserved sequences (domains) of efflux pumps or identifying unannotated/misannotated genes of similar function. It is unclear which trait(s) of these genes contributes to being targeted by this primer.

To illustrate less-desirable unintended primer binding, we discuss the primers targeting genes of the SMR superfamily (Fig. 3). This superfamily is represented by genes from P. putida, *G. subterraneus*, *Acetobacterium woodii*, and B. subtilis for a total of eight distinct target genes. Out of these eight targets, the primers for B. subtilis *qacE1* (QU35_18255) and *ebrA* (QU35_09520) have no unintended binding locations. The upstream primers for B. subtilis *emrE* (QU35_06845) and P. putida *emrE* (PP_4930) each have a single unintended binding location with 90.0 to 99.9% identity, targeting a noncoding region in *G. subterraneus* (GSUB:834814) and a *betA* gene in P. putida (PP_3383), respectively. Six of the remaining primers have unintended targets with 80.0 to 89.9% identity, targeting 24 different genes encoding proteins ranging from hypothetical proteins (QU35_15730, Tmz1t_0375, and Tmz1t_0977) to transporter permeases (QU35_06380, QU35_21320, and QU35_18115), a putative symporter (PP_3247), polymerases (Awo_c25450 and Tmz1t_3813), isomerases (DvMF_3022 and QU35_19985), and regulators and stress proteins (QU35_03275, DvMF_0255, PP_1269, and PP_3526). The lower percent identity matches (80.0 to 89.9%) do not target genes of a predictable or specific function but, rather, the targets share little similarity despite some membrane-associated proteins being detected. The unintended efflux genes detected are not of the same superfamily as the intended targets but rather are of the ABC superfamily. Interestingly, primers designed as part of this work to target the ABC superfamily, for which there is only a single target (*lmrA* from B. subtilis; QU35_01610), have zero unintended binding locations.

Finally, we discuss the unintended targeting of three different primers, two of which were constructed with degenerate bases and intended to target multiple sequences in different species. The two degenerate primers are the upstream *acrB/mexD* targeting both genes in P. putida and the downstream primer targeting *acrB* in T. aromatica and *mexD* in P. putida. The single target primer is the downstream *acrB* for P. putida, which complements the P. putida *acrB/mexD* primer. For the intended targets, all primers anneal with 100% identity. Due to the degenerate nature of two of these primers, there is a significant increase in the unintended binding targets compared to other primer sets (Fig. 4). Of note is the complexity of this figure, which has been intentionally retained to highlight the issues of the unintended primer binding locations as the gene targets become more complicated. Interestingly, the unintended binding locations of the upstream *acrB/mexD* primer are exclusively between 90.0 and 99.9% sequence identity, while the P. putida downstream *acrB* primer has a mix of high percent identity and low percent identity binding sites. Some of the recurring unintended targets are the other *mex* genes, specifically *mexB* and *mexF*, both of which are detected by the *acrB/mexD* upstream and downstream primers with identities of 90.0 to 99.9% (with the exception of *mexB*, which unintentionally has 100% identity with the T. aromatica/P. putida *mexD* downstream primer). The T. aromatica/P. putida *mexD* downstream primer also has 100% identity matches with *mexF* (PP_3426) and the gene encoding a putative RND transporter (PP_0906) (Fig. 4). Many of the unintended targets of this degenerate downstream primer are genes coding for hypothetical proteins and all have annealing temperatures of 50.0 to 59.9°C (a minimum of 5°C below the melting temperature of the intended target). Of the unintended targets of the upstream P. putida *acrB/mexD* primer, two are hydrophobe/amphiphile efflux-1 (HAE1) genes (DvMF_0036 and Tmz1t_0505), one encodes an uncharacterized RND transporter in *G. subterraneus* (GSUB_04250), and the final target is the *ttgB* gene (PP_1385) in P. putida, which codes for a probable membrane efflux pump transporter protein.

This primer set illustrates the ability of the primers designed from a multiple-sequence alignment (MSA) of multiple genes (11 annotations in total) to target and locate other, similar genes both within the intended target species and in other, unrelated species owing partially to the degenerate bases present. Most hits are located within the two intended species, with only two hits occurring outside, genes encoding HAE1 in *D. vulgaris* and an RND transporter in *G. subterraneus*. It is important to note that RND primers are the only primers to have both upstream and downstream primers binding simultaneously to the same target, potentially resulting in unintended PCR amplicons. This occurs in four different genes: *mexF* (PP_3426), *ttgB* (PP_1385), HAE1 (Tmz1t_0505), and *mexB* (PP_3456) (Fig. 4). A fifth gene (encoding a putative RND transporter, PP_0906) is targeted by multiple primers; however, this gene is only targeted by downstream primers which bind to the identical location and thus cannot produce a PCR amplicon. Perhaps unsurprisingly, the primers targeting *mexD* in P. putida also bind to and would produce a PCR amplicon on *mexB* (PP_3456) and *mexF* (PP_3426), suggesting that these genes have a homologous domain which influenced the MSA of the *mexD* genes. The other two unintended targets with multiple primer attachments target a *ttgB* (PP_1385) gene in P. putida, which encodes a probable efflux pump, and an HAE1 gene in T. aromatica (Tmz1t_0505), encoding a protein that belongs to the acriflavine resistance protein B family (efflux pump) according to KEGG. It becomes clear from this example that the degenerate bases allow for an increase in unintended target locations and, unlike the other examples, these primers will produce actual PCR amplicons, disrupting any potential qPCR or downstream analyses. Although these unintended amplicons can be accounted for and removed using bioinformatics during sequencing applications, in qPCR applications these products, especially if they are of similar size to the intended product (or more specifically produce amplicons with similar melting points), can produce false positives without being able to distinguish between the intended and unintended products. Though the focus here was to design primers for PCR to determine presence and abundance of target genes in a community, the primers could be used in a parallel workflow for sequencing.

To address an issue resulting from our primer design A approach (i.e., designing with degenerate bases), we developed an alternative method (primer design B) to create primer sets which preferentially target different locations (i.e., potentially unconserved locations) but prioritize maintaining identical amplicon sizes across all intended targets. As illustrated with the RND primers, design A can lead to an increase in unintended binding locations due to the exponential increase in primer sequences. Each degenerate base increases the number of unique sequences present in the primer mix and may create a primer which has no intended target sequence, meaning the chance of unintended binding increases. To illustrate, take the example outlined in Table 3 for the upstream primer targeting the *acrB* and *mexD* genes in P. putida.

**TABLE 3:** Example of a comparison of intended and unintended sequences when degenerate bases are used

Type of primer	Sequence^*a*^
Desired sequence A	TGG CGG CGC AGT ACG AAA GC
Desired sequence B	TGG TGG CGC TGT ACG AAA GC
Resulting degenerate primer	TGG **Y**GG CGC **W**GT ACG AAA GC
All combinations	TGG **C**GG CGC **A**GT ACG AAA GC
	TGG **C**GG CGC **T**GT ACG AAA GC
	TGG **T**GG CGC **A**GT ACG AAA GC
	TGG **T**GG CGC **T**GT ACG AAA GC

a Degenerate (boldface) base codes: Y = C, T; W = A, T; R = A, G; S = G, C; M = A, C.

From this simple example using two degenerate bases coding for only two nucleotides each, the resulting degenerate primer consists of a mixture of four primers, two of which do not code for any intended sequence. Rather, a more precise approach is to treat each gene as its own template, design primers to create the same amplicon size, and have similar melting temperatures and subsequently combine all the upstream primers into a single mixture in equal proportions. In this way, the number of primers is kept to a minimum and every primer has a desired target. An alternative to degenerate bases is the use of inosine; however, this will not always improve primer function (42). Based on this previous study, the use of degenerate bases improves the broad range detection of a target gene, but the primers suffer from nonspecific amplification, dimerization, and primer slippage. The use of inosine in place of degenerate bases on occasion improved primer detection, but this was not universally the case across all their primer sets (42). Correct use of degenerate bases or inosine must be assessed on a case-by-case basis.

The unintended specificity of these primers is of note when considering that all the primers are 18 to 23 nucleotides in length and therefore target a sequence of six or seven amino acids. It must be understood that not all MDREP genes would be detected using this approach, but it could provide a means of improving our understanding of conserved regions and, as this work illustrates, the conserved amino acid region may be as short as six or seven amino acids and still provide relatively high accuracy for gene identification. It is clear from this that even a six-amino-acid sequence can have evolutionary pressure to convey similarity in overall protein structure and/or function.

Using approach B (i.e., mixing each unique primer together without degenerate bases) allows the “universal” primer mix to always be in flux and be improved as new primers get added, until such point as the number of primers present negatively affects a specific primer’s ability to bind the correct sequence. A consideration with approach B is that because the primers do not always target conserved regions, the primers may have unintended targets elsewhere in the genome(s), entirely unrelated to the target sequence. As a result, and as is good practice, primer sequences developed using either approach should be tested against the target genome(s) and all unintended binding locations should be identified and accounted for during PCR protocol development.

### MDREP primers compared to universal 16S rRNA primers.

In contrast to the successful universal primer designs targeting the 16S rRNA gene, the issues discussed here highlight the difference between targeting core genes (essential for replication), character genes (those defining the type of metabolism), and accessory genes (genes which may provide improved fitness under specific conditions). As we improve techniques and apply genetic screening to more and more health (e.g., infection, disease, etc.) and economically significant (e.g., agriculture, bioremediation, etc.) issues, the more relevant genetic targets we will discover. Logically, the more specific the target, the more meaningful the presence/absence and quantity become but the further away from the core genes (moving toward characteristic and accessory genes) we must move. Accessory genes (and to a lesser extent characteristic genes) have less evolutionary pressure on them, which allows for higher rates of mutation and variability on the nucleotide level. Additionally, as many accessory genes are or can be located on mobile genetic elements, they become subject to the nucleotide biases and codon usages present in their current host. While conserved genes may have variable regions flanked by conserved regions (e.g., 16S rRNA genes) which can be targeted to facilitate primer design, nonconserved genes may not reliably have conserved regions, forcing primers to target less-conserved regions more susceptible to mutations and variations. Furthermore, the mechanism of the movement may affect the gene’s availability or expression levels. The fitness of an accessory gene is dependent on environmental pressures and is subject to pulses of challenge, such as short periods of exposure to biocide, as is typical for pipeline antimicrobial treatments or antibiotic courses.

For comparisons, the percent identities of the target genes were calculated using the multiple-sequence alignment (MSA) for each respective gene. The scores were calculated using the equation

(1) Percent identity=(matches×100)/length of MSA ( including gaps)

Each MSA was calculated using the conditions described in Materials and Methods. Only a single representative gene for each superfamily was selected as an indication of the variability within that superfamily. The average scores of the sequence identities are listed in Table 4, and a complete list of each gene’s scores is provided in Tables S2 to S6 in the supplemental material. The ABC superfamily has been omitted due to low gene counts across the six representative species.

**TABLE 4:** Average scores of percent identities for genes chosen to represent four superfamilies^*a*^

Superfamily	Gene	MSA length (bp)	Gene count	Percent identity score	SD
16S rRNA		1,595	32	84.71	4.32
MFS	*qacA/emrB*	1,630	6	58.13	6.15
SMR	*emrE*	1,137	3	49.99	21.23
MATE	*norM*	1,521	3	67.63	1.11
RND	*acrB*	3,990	11 [9]	43.99 [52.71]	19.21 [5.71]

a MSA, multiple-sequence alignment; SD, standard deviation. Modified values, obtained after removal of two significantly shorter annotations, are provided in brackets.

From the percent identity scores, it is obvious the 16S rRNA gene is more conserved than any of the efflux pump genes. The highest score belongs to the *norM* gene, which among the annotated copies had very high percent identity (67.63%), while *acrB* and *emrE* had low scores (43.99 and 49.99%, respectively). The *acrB* score is skewed from two annotated copies being significantly shorter than the MSA, producing percent identity scores of 5.26% and 4.24%. Removing these two copies produced a score of 52.71% ± 5.71%, which brings the score for *acrB* more in line with *qacA/emrB* and *emrE* scores.

These scores reflect the relative simplicity of designing primers for highly conserved core genes, while nonessential, accessory genes are significantly more difficult due their more plastic nature. While they are less homologous, the identity scores suggest it is possible to design universal primers for these peripheral genes. However, these genes do require a different approach for primer design. Using approach A, which employs multiple sequence alignments, more conserved regions can be identified to target, whereas using approach B, which employs unique primer sequences (avoiding the use of degenerate bases), results in a primer mixture with higher accuracy and specificity. Where approach A may have a higher universality to the genes detected, the use of degenerate bases requires a more in-depth investigation into unintended hits. In contrast, approach B will provide more specific, targeted results with higher confidence, but the creation of universal mixes through the mixture of targeted primers detects less-diverse targets.

The plasticity of nucleotide sequences of the accessory genes results from the improved fitness benefits occurring only under specific conditions that are not always present. This allows for mutations to occur that would otherwise be impossible in core or character genes. Due to the nonconserved nature of MDREP genes and their mobility through and across genomes, the abundance and variance on the genetic level are erratic and unpredictable. The presence of the same gene in two different species does not allow for the determination of the direction of flow or origin of the gene. The *in silico* approach described here has the potential to be exploited to investigate evolutionary branching in these genes and in the case of MDREP genes, potentially shedding light on how these genes are selected for and allow for the identification of other clinically relevant efflux pumps yet to be discovered.

As with the 16S rRNA example, successful primer sets may be used for sequencing the intergenic sequences. The degree to which this sequencing will allow for more accurate gene annotation or phylogenetic assignment will be dependent on the depth of sequencing of a given target gene. The primers then also become useful for other, related wet-lab techniques, such as reverse transcriptase PCR (RT-PCR) or FISH.

### Control group binding.

The primers used as a control to assess the primer design methods target the gene encoding 3-isopropylmalate dehydratase subunit LeuC (ECK0074), which has been characterized as nonessential according to the Profiling of E. coli Genome (PEC) database (http://www.shigen.nig.ac.jp/ecoli/pec/). This gene represents an example of a characteristic gene, which is one susceptible to mutations but not to the same degree as the MDREP genes, nor is it expected to be as mobile. Upon searching the six genomes for the terms “LeuC” and “3-isopropylmalate,” seven total annotated genes were collected and used to create an MSA (other genes were found but attributed to the small subunit, *leuD*). Details of the primers used in this example are shown in Table 5. Based on the MAFFT default settings of Benchling, the MSA was split into two sequences to improve overall alignment scores. MSA1 consisted of both A. woodii sequences and those of D. vulgaris and G. subterraneus, while MSA2 comprised sequences of B. subtilis, P. putida, and T. aromatica. A summary of the number of gene hits using these primers is in Table 6, and a full list of gene hits can be found in Table S7. Table 6 shows that the primers containing degenerate bases have more unintended primer binding locations, especially the MSA1 upstream primer. A significant amount of the unintended hits for all *leuC* primers in the 90.0 to 99.9% identity range are attributable to *leuC* genes from the other strains (17/27), indicating that both MSAs were successful in identifying highly conserved regions within the *leuC* gene. At the 80.0 to 89.9% identity range, the primer hits become far less specific, detecting noncoding regions (5/41) or unannotated/hypothetical/unknown functional genes (4/41) as frequently as *leuC* (5/41). The degenerate primers detected fewer hypothetical proteins than the MDREP primers, which we attribute to the higher rate of correct *leuC* annotation, owing to its more conserved status compared to the MDREP primers.

**TABLE 5:** Details of *leuC* primer design example

Genome	No. of *leuC* annotated copies	MSA no.^*b*^	Upstream primer sequence (*T_m_* °C)^*a*^	Downstream primer sequence (*T_m_* °C)
*A. woodii*	2	1	5′- cat acc tgt act cat ggg gct t (55.6)	5′- ccg gcc tcg atc gcc ata tt (55.1)
*A. woodii*	2	1	5′- cat acc tgt acc tat ggt gcg c (57.1)	5′- ccg gct tca atg gcc ata tt (58.9)
B. subtilis	1	2	5′- tcg aag ttg cgg tta gag gt (55.4)	5′- atc ggt tcc tgc aca aa (50.6)
*D. vulgaris*	1	1	5′- cac acc tgc acc tac ggg ggg c (65.6)	5′- ccc gcc tcg atg gcc atg tt (61.7)
*G. subterraneus*	1	1	5′- cac acc tgt acc cat ggg gcc t (62.3)	5′- ccg gct tcg atg gcc atg tt (59.9)
P. putida^*b*^	1	2	5′- tcg aag ttg cgg ttg gag gt (58.4)	5′- atc ggc tcg tgc acc aa (56.2)
T. aromatica	1	2	5′- tcg aag ttg cgg ttc gag gt (58.5)	5′- atc ggc tcg tgc acc aa (56.2)
MSA 1	NA	1	5′- caY acc tgY acY YaY ggK gSK Y (51.1–66.0)	5′- ccS gcY tcR atS gcc atR tt (51.8–57.9)
MSA 2	NA	2	5′- tcg aag ttg cgg ttV gag gt (51.8–53.8)	5′- atc ggY tcS tgS acM aa (44.6–49.5)

a Degenerate base codes: S = C/G; Y = C/T; M = A/C; K = G/T; R = A/G; V = not T; MSA, multiple-sequence alignment; *T_m_*, thermal denaturation point.

b Based on MSA, the orientation of the primers is reverse on this sequence.

**TABLE 6:** Summary of primer-binding hits of the *leuC* primers

Intended target sequence (annotation no.)	Upstream primer hit no.			Downstream primer hit no.		
	100% identity	99.9–90.0% identity	89.9–80.0% identity	100% identity	99.9–90.0% identity	89.9–80.0% identity
MSA1	4	2	20	4	7	0
MSA2	3	2	3	4	0	0
*A. woodii* -1 (Awo_c02850)	1	1	0	1	1	0
*A. woodii* -2 (Awo_c13960)	1	0	0	1	1	0
*B, subtilis* (QU35_15365)	1	2	2	1	0	0
*D. vulgaris* (DvMF_1792)	1	0	0	1	2	0
*G subterraneus* (GSUB_10900)	1	0	1	1	3	12
P. putida (PP_0596)	1	4	1	3	0	0
T. aromatica (Tmz1t_3071)	1	2	2	2	0	0

### Applications in metagenomic data sets.

A major consideration of primer design is application to metagenomic data sets. Due to the diversity and size of these data sets, they are typically poorly annotated, with thousands of predicted genes with no known function or hypothetical proteins; thus, searching for a specific gene using a name or annotation has the potential to return false-negative results, or even false positives due to misannotation. Thus, it is suggested that primers be designed based not on any annotations from a metagenomic data set but rather by using a type strain of a species expected to be present in the metagenomic environment of interest. This will improve the primer accuracy but has the limitation of targeting a narrower subset of the desired target gene. For highly conserved genes, this is less of an issue, but for highly mobile, nonconserved genes such as the MDREPs used here, this may further reduce the number of primer-detected genes. The more “universal” a primer set is, i.e., the more sequences used to compile the MSA used in primer design, the more potent the detection rate would become.

### Considerations and limitations.

While this approach endeavors to reduce the amount of wet-lab troubleshooting by anticipating pitfalls such as loss of primer efficiency through off-target binding, there will always be some degree of benchtop troubleshooting required. This work is not meant to replace or eliminate wet-lab work but rather to improve primer design and reduce the well-known issue of dependence on annotation databases (43). It is important to keep in mind the specific end goal of the primers (e.g., qPCR, amplicon sequencing, key pathogen or metabolism potential identification) and how that will affect parameters of the primer design. Even when targeting the same gene, a different end goal may require separate primers.

Here, initial *in vitro* validation of some of the more complicated primers employing degenerate bases has been done against P. putida and T. aromatica pure-culture DNA and DNA collected from a selection of field samples of fresh surface water samples with various biocide treatments (untreated, bronopol, glutaraldehyde, DBNPA, and quaternary ammonium compounds). The PCR products were separated using 1.5% agarose gels run at 100 V for 45 min (Fig. S.1 to Fig. S.4 in the supplemental material). The primer sets chosen were 16S rRNA for field template validation (amplicon size: 292) (Fig. S.1), *qacA* from P. putida (*P.puti* qacA1/3_U and *P.puti* qacA1_D; amplicon size: 198) (Fig. S.2), *mexD* from P. putida (*P.puti* acrB/mexD_U and *T.aro* acrB2/*P.puti* mexD_D; amplicon size: 187), *acrB2* from T. aromatica (*T.aro* acrB2_U and *T.aro* acrB2/*P.puti* mexD_D; amplicon size: 187) (Fig. S.3), and *acrB* from P. putida (*P.puti* acrB/mexD_U and *P.puti* acrB_D; amplicon size: 187) (Fig. S.4). These gels show that field DNA, regardless of biocide treatment, is suitable for PCR as the 16S rRNA primers were all successful (Fig. S.1) but that *qacA* was not detectable in these samples (Fig. S.2). The *qacA1* primers have two unintended products against T. aromatica (∼400 and ∼750 bp) but the single intended product against P. putida. The primer sets targeting *mexD* and *acrB2* (Fig. S.3) have identical amplicon products when used against P. putida and T. aromatica, suggesting that these primers, which employ degenerate bases and share a downstream primer (*T.aro* acrB2/*P.puti* mexD_D), are unable to distinguish between the two intended targets at the given annealing temperature (58°C). The primer set targeting *acrB* from P. putida illustrates how the PCR cycling conditions must still be properly tuned, as there is a single product when used with an annealing temperature of 63°C against P. putida and T. aromatica (Fig. S.4, lane 5) but many unintended products against P. putida when used with an annealing temperature of 58°C (Fig. S.4, lane 13). All these gels indicate that none of the samples have P. putida or T. aromatica in them, or any target sequences of high enough similarity to be detected by these primer sets. From the pure-culture DNA templates, it is clear the primers work as intended (when used with appropriate annealing temperatures) but were unable to detect these targets in the field samples. To further validate these primers, the PCR assays were performed on samples with the field DNA samples spiked with 1% total DNA concentration of both P. putida and T. aromatica genomic DNA (Fig. S.5 and S.6). When the samples were spiked with the genomic DNA of the pure cultures, the expected bands were produced for every sample (with the exception of the NTC), showing that these primers are still able to function in these field samples, detecting sequences added at 1% of the total DNA concentration.

### Lessons learned.

An overlying issue with primer design attempts such as this one is the often-poor annotation quality of our genomic data libraries, particularly for environmental (or more generally any nonclinical) species. The abundance of putative, predicted, or hypothetical proteins severely limits the ability to accurately find related genes to design primers for specific genes. To alleviate this issue, primers should be designed using a well-annotated genome which is likely to be present in the environment where the primers will ultimately be used. In this way, the primers can be designed with higher confidence and, as shown here, can be used to probe genomes with lower annotation quality and aid in identifying the desired targets there.

As with all scientific endeavors, it behooves the scientist to keep in mind the ultimate goal of the primers. Here, we attempted to design primers for the same genes across six different genomes to eventually combine them into a “universal” primer mix for the desired target. In these situations, unintended binding becomes a larger issue because the amount of a specific primer is already reduced with respect to the final primer concentration, and any off-site binding could result in false negatives during PCR amplification. If the objective is to probe a mixed sample for the presence of a specific gene (e.g., for clinical or environmental screening), the use of degenerate primers becomes risky as it may lead to false positives or negatives (should the mixture of primers be too complex and the competitive binding of the primers prevent correct primer binding). Alternatively, in exploratory science such as the attempt explained here, the degenerate primers can increase the ability of the primers to detect additional genes not identified in the annotations. There is the potential that many of the primer-selected genes coding for hypothetical proteins actually represent efflux pumps of some nature, and thus we can add additional evidence toward these predicted proteins. This would then require a more targeted investigation of the genes identified in this manner, such as comparing the amino acid sequences of the predicted genes to known proteins and determining whether they truly would encode efflux pumps.

Unlike for the 16S rRNA gene family, designing universal primers for mobile, accessory genes is particularly difficult. Of note from this approach are the utility and the potential of *in silico* analysis of primers designed for less-conserved genes and their potential to aid or facilitate improved annotation in less-studied organisms.

The choice concerning which of the two primer design approaches should be employed becomes a decision based on the percent identity or conservation of the nucleotide sequences of the target genes. To facilitate the decision, percent identity scores should be calculated for all annotated copies of the desired target gene. From the 16S rRNA and MDREP gene examples shown here, we suggest a cutoff value of 75%, where identity scores above 75% should use primer design A (employing MSA and degenerate bases) while identity scores below 75% would be more effective if primers were designed using design B (unique sequences with variable binding locations). This should reduce the amount of unintended binding locations and overall improve the efficacy of primer binding in PCR and qPCR applications.

In conclusion, this work illustrates the benefits and shortcomings of two different primer design approaches. First, the use of multiple-sequence alignments (MSAs) to locate conserved regions of the nucleic acid lends itself toward creating primers with degenerate bases and ensures uniform PCR amplicon size. With the degenerate bases, these primers are more likely to have unintended binding and lower primer efficiency during thermocycling. However, these primers are more likely to reveal the presence of the desired gene(s) in poorly annotated genomes when using an *in silico* method.

The second primer design approach creates primers from individual genes without targeting conserved regions of the MSA while controlling for melting temperature and amplicon size to ensure all primers designed in this way are compatible. This approach allows for more-stringent thermocycling conditions and reduces the amount of unintended primer binding locations (thus improving detection rates *in vitro*), but this approach is less likely to reveal similar or identical genes in mixed environments.

Overall, this work highlights how extremely important it is to appreciate the false discovery rates resulting from the chosen primer design approach and the subsequent ramifications in interpretations when it comes to defining one’s goal.

## MATERIALS AND METHODS

### *In silico* gene identification.

Six bacteria were chosen to represent a highly simplified mixed-species community as may be identified in a microbiologically influenced corrosion environment. The chosen strains all had fully sequenced genomes available on the Joint Genome Institute (JGI) website (https://img.jgi.doe.gov/cgi-bin/m/main.cgi). The IMG Genome ID numbers for the representative genomes are as follows: Acetobacterium woodii (2512047040), Bacillus subtilis (2511231064), Desulfovibrio vulgaris (637000096), Geoalkalibacter subterraneus (2593339207), Pseudomonas putida (641522645), and Thauera aromatica (2791355019).

The full genome sequences were imported from JGI to a Web-hosted sequence alignment tool (Benchling) for gene sequence alignments. Multidrug resistance efflux pump genes were identified through searching directly for their gene names and abbreviations or key words, including (but not exclusively) multidrug, efflux, transporter, outer membrane, inner membrane, and resistance. A recognized challenge was that not all genomes were equally annotated. For example, the genome of P. putida has a more complete annotation than a more environmentally relevant species such as *D. vulgaris*. Once identified, all copies of each gene were clustered and a nucleic acid multiple-sequence alignment (MSA) was performed with no template sequence and using the MAFFT alignment algorithm with default conditions (maximum iterations: 0; tree rebuilding number: 2; gap open penalty: 1.53; gap extension penalty: 0.0; and no adjust direction). MSAs were used to identify regions of high nucleotide percent identities across all annotated genes of the same name. These regions were preferentially used for primer design as described below. No specific percent identity was used to identify regions to target for primer design; rather, regions were selected to minimize the number of mismatches while still maintaining amplicon sizes, as described below.

### Primer design approaches.

**(i) Primer design approach A (traditional).** Using the MSA, homologous regions of the nucleotide sequences were identified and used as targets for primer binding. All primer sequences and details are reported in Table 2. Using the Benchling platform, primers were all designed to be 18 to 23 bp in length and create PCR amplicons of 180 to 240 bp in length to facilitate quantitative PCR (qPCR) analysis as a downstream application. The GC content was targeted to be 50% but exceptions were made, allowing the GC content to reach a maximum of 80% (Table 2) to accommodate the target regions. Although efforts were made to maintain the same melting temperature (±5°C) between the upstream (forward) and downstream (reverse) primers for all primer pairs of a specific gene, priority was given to optimizing the melting temperatures of a specific pairing (albeit this rule had to be stretched on occasion as well). The position along the MSA which matched all these conditions was used to create primers and, when required, primers were designed with degenerate bases to ensure they targeted the desired locations of the MSA.

**(ii) Primer design approach B (novel).** As a result of the limitations of primer design approach A, a unique approach was developed to more efficiently target genes annotated the same but with a lower percent identity. Again, using the Benchling platform, primers were designed to be 18 to 23 nucleotides in length, produce an amplicon of 180 to 240 bp, and have the same melting temperatures (± 5°C) between the upstream and downstream primer pairs. To avoid the use of degenerate bases, the primer positioning against the MSA was more flexible, allowing the binding locations for the primer pairs to drift while maintaining identical amplicon size. Using this approach, the amplicon size was a higher priority than annealing location, thereby preventing issues with downstream analysis across different primer pairs targeting the same genes in different genomes.

### Primer binding testing.

Intended and unintended primer binding locations were identified using the following *in silico* conditions on the Benchling platform. The SantaLucia 1999 algorithm was selected by default, primer binding parameters were a minimum of 18 matched bases, a maximum of three mismatches total with no consecutive mismatches was allowed, and annealing temperatures were between 30 and 100°C. The three allowed mismatches result in a minimum percent identity of >80%. Due to the binding algorithm of Benchling’s primer binding, primers with mismatched 5′ ends had to be manually removed from the pool (no mismatches were allowed on the 3′ end by default). A full list of the binding locations for all MDREP primers is available in Table S1. To identify primer binding locations, the locus tag of the gene was collected from Benchling and the NCBI GenBank files were queried to identify the product name. In cases where no product name was provided for a specific locus tag, the protein ID was investigated and a gene identity was assigned from the region name.

### Control targets.

To further validate these approaches, primers were designed against a control group distinct from the MDREP class of genes used here. The control gene chosen was 3-isopropylmalate dehydratase large subunit (*leuC*), of which there are two annotated copies in *A. woodii* and one copy each in B. subtilis, *D. vulgaris*, *G. subterraneus*, P. putida, and T. aromatica. Primers were designed using the MSAs of the seven annotated copies, which were subsequently split into two distinct MSAs based on overall alignments. From these MSAs, primers with degenerate bases were designed, followed by unique primers for identical locations but with no degenerate bases so each individual sequence has a unique primer set. Identical primer-binding conditions were used as before with the exception that binding was allowed to be 17 bases while maintaining the maximum number of three mismatches, which still produces primers with a minimum of 80% identity on primers of 20 bases in length.

### 16S comparison.

A primer set targeting 16S rRNA has been selected as a tool for comparison against well-established universal primer sets (2). This primer set employs degenerate bases and consists of the upstream primer (5′-GTGCCAGC**M**GCCGCGGTAA) and the downstream primer (5′-GGACTAC**HV**GGGT**W**TCTAAT), with annealing temperatures of 62.6°C and 48.9°C, respectively. These primers are designed to target the conserved nucleotide regions flanking the V4 region of the 16S rRNA gene and produce an amplicon of 292 nucleotides (2).

## Supplementary Material

Supplemental file 1

## References

[1] Nhung PH, Ohkusu K, Miyasaka J, Sun XS, Ezaki T 2007 Rapid and specific identification of 5 human pathogenic Vibrio species by multiplex polymerase chain reaction targeted to dnaJ gene. Diagn Microbiol Infect Dis 59:271–275. doi:10.1016/j.diagmicrobio.2007.05.016. 17614235

[2] Caporaso JG, Lauber CL, Walters WA, Berg-Lyons D, Lozupone CA, Turnbaugh PJ, Fierer N, Knight R 2011 Global patterns of 16S rRNA diversity at a depth of millions of sequences per sample. Proc Natl Acad Sci U S A 108:4516–4522. doi:10.1073/pnas.1000080107. 20534432 PMC3063599

[3] Leloup J, Quillet L, Oger C, Boust D, Petit F 2004 Molecular quantification of sulfate-reducing microorganisms (carrying dsrAB genes) by competitive PCR in estuarine sediments. FEMS Microbiol Ecol 47:207–214. doi:10.1016/S0168-6496(03)00262-9. 19712335

[4] Horz HP, Vianna ME, Gomes BPFA, Conrads G 2005 Evaluation of universal probes and primer sets for assessing total bacterial load in clinical samples: general implications and practical use in endodontic antimicrobial therapy. J Clin Microbiol 43:5332–5337. doi:10.1128/JCM.43.10.5332-5337.2005. 16208011 PMC1248440

[5] Lass-Florl C, Aigner J, Gunsilius E, Petzer A, Nachbaur D, Gastl G, Einsele H, Loffler J, Dierich MP, Wurzner R 2001 Screening for Aspergillus spp. using polymerase chain reaction of whole blood samples from patients with haematological malignancies. Br J Haematol 113:180–184. doi:10.1046/j.1365-2141.2001.02744.x. 11328298

[6] Hebart H, Löffler J, Meisner C, Serey F, Schmidt D, Böhme A, Martin H, Engel A, Bunje D, Kern WV, Schumacher U, Kanz L, Einsele H 2000 Early detection of *Aspergillus* infection after allogeneic stem cell transplantation by polymerase chain reaction screening. J Infect Dis 181:1713–1719. doi:10.1086/315435. 10823773

[7] Read SJ, Kurtz JB 1999 Laboratory diagnosis of common viral infections of the central nervous system by using a single multiplex PCR screening assay. J Clin Microbiol 37:1352–1355. doi:10.1128/JCM.37.5.1352-1355.1999. 10203485 PMC84773

[8] Baker GC, Smith JJ, Cowan DA 2003 Review and re-analysis of domain-specific 16S primers. J Microbiol Methods 55:541–555. doi:10.1016/j.mimet.2003.08.009. 14607398

[9] Yang B, Wang Y, Qian P-Y 2016 Sensitivity and correlation of hypervariable regions in 16S rRNA genes in phylogenetic analysis. BMC Bioinformatics 17:135. doi:10.1186/s12859-016-0992-y. 27000765 PMC4802574

[10] Chakravorty S, Helb D, Burday M, Connell N, Alland D 2007 A detailed analysis of 16S ribosomal RNA gene segments for the diagnosis of pathogenic bacteria. J Microbiol Methods 69:330–339. doi:10.1016/j.mimet.2007.02.005. 17391789 PMC2562909

[11] Pinto AJ, Raskin L 2012 PCR biases distort bacterial and archaeal community structure in pyrosequencing datasets. PLoS One 7:e43093. doi:10.1371/journal.pone.0043093. 22905208 PMC3419673

[12] Suzuki MT, Giovannoni SJ 1996 Bias caused by template annealing in the amplification of mixtures of 16S rRNA genes by PCR. Appl Environ Microbiol 62:625–630. doi:10.1128/AEM.62.2.625-630.1996. 8593063 PMC167828

[13] Brown D, Demeter M, Turner RJ 2019 Prevalence of multidrug resistance efflux pumps (MDREPs) in environmental communities, p 545–557. *In* Das S, Dash HR (ed), Microbial diversity and infectious diseases, 1st ed Elsevier Inc, London, United Kingdom.

[14] Piddock LV 2006 Multidrug-resistance efflux pumps—not just for resistance. Nat Rev Microbiol 4:629–636. doi:10.1038/nrmicro1464. 16845433

[15] Alekshun MN, Levy SB 2007 Molecular mechanisms of antibacterial multidrug resistance. Cell 128:1037–1050. doi:10.1016/j.cell.2007.03.004. 17382878

[16] Nikaido H, Pagès JM 2012 Broad-specificity efflux pumps and their role in multidrug resistance of Gram-negative bacteria. FEMS Microbiol Rev 36:340–363. doi:10.1111/j.1574-6976.2011.00290.x. 21707670 PMC3546547

[17] Nikaido H 1998 Antibiotic resistance caused by Gram‐negative multidrug efflux pumps. Clin Infect Dis 27:S32–S41. doi:10.1086/514920. 9710669

[18] Spengler G, Kincses A, Gajdács M, Amaral L 2017 New roads leading to old destinations: efflux pumps as targets to reverse multidrug resistance in bacteria. Molecules 22:468–492. doi:10.3390/molecules22030468. PMC6155429 28294992

[19] Schuster S, Vavra M, Kern WV 2016 Evidence of a substrate-discriminating entrance channel in the lower porter domain of the multidrug resistance efflux pump AcrB. Antimicrob Agents Chemother 60:4315–4323. doi:10.1128/AAC.00314-16. 27161641 PMC4914648

[20] Lewinson O, Adler J, Sigal N, Bibi E 2006 Promiscuity in multidrug recognition and transport: the bacterial MFS Mdr transporters. Mol Microbiol 61:277–284. doi:10.1111/j.1365-2958.2006.05254.x. 16856936

[21] Poole K 2007 Efflux pumps as antimicrobial resistance mechanisms. Ann Med 39:162–176. doi:10.1080/07853890701195262. 17457715

[22] Chapman JS 2003 Biocide resistance mechanisms. Int Biodeterior Biodegrad 51:133–138. doi:10.1016/S0964-8305(02)00097-5.

[23] Harbottle H, Thakur S, Zhao S, White DG 2006 Genetics of antimicrobial resistance. Anim Biotechnol 17:111–124. doi:10.1080/10495390600957092. 17127523

[24] Stokes HW, Gillings MR 2011 Gene flow, mobile genetic elements and the recruitment of antibiotic resistance genes into Gram-negative pathogens. FEMS Microbiol Rev 35:790–819. doi:10.1111/j.1574-6976.2011.00273.x. 21517914

[25] Broaders E, Gahan CGM, Marchesi JR 2013 Mobile genetic elements of the human gastrointestinal tract: potential for spread of antibiotic resistance genes. Gut Microbes 4:271–280. doi:10.4161/gmic.24627. 23651955 PMC3744512

[26] El Salabi A, Walsh TR, Chouchani C 2013 Extended spectrum β-lactamases, carbapenemases and mobile genetic elements responsible for antibiotics resistance in Gram-negative bacteria. Crit Rev Microbiol 39:113–122. doi:10.3109/1040841X.2012.691870. 22667455

[27] Karkman A, Do TT, Walsh F, Virta MPJ 2018 Antibiotic-resistance genes in waste water. Trends Microbiol 26:220–228. doi:10.1016/j.tim.2017.09.005. 29033338

[28] Zhang T, Zhang XX, Ye L 2011 Plasmid metagenome reveals high levels of antibiotic resistance genes and mobile genetic elements in activated sludge. PLoS One 6:e26041. doi:10.1371/journal.pone.0026041. 22016806 PMC3189950

[29] Marti E, Variatza E, Balcazar JL 2014 The role of aquatic ecosystems as reservoirs of antibiotic resistance. Trends Microbiol 22:36–41. doi:10.1016/j.tim.2013.11.001. 24289955

[30] Guo J, Li J, Chen H, Bond PL, Yuan Z 2017 Metagenomic analysis reveals wastewater treatment plants as hotspots of antibiotic resistance genes and mobile genetic elements. Water Res 123:468–478. doi:10.1016/j.watres.2017.07.002. 28689130

[31] Wang Z, Zhang XX, Huang K, Miao Y, Shi P, Liu B, Long C, Li A 2013 Metagenomic profiling of antibiotic resistance genes and mobile genetic elements in a tannery wastewater treatment plant. PLoS One 8:e76079. doi:10.1371/journal.pone.0076079. 24098424 PMC3787945

[32] Saier MH, Jr, Paulsen IT 2001 Phylogeny of multidrug transporters. Semin Cell Dev Biol 12:205–213. doi:10.1006/scdb.2000.0246. 11428913

[33] Poole K 2001 Multidrug resistance in Gram-negative bacteria. Curr Opin Microbiol 4:500–508. doi:10.1016/s1369-5274(00)00242-3. 11587924

[34] Altenberg GA 2004 Structure of multidrug-resistance proteins of the ATP-binding cassette (ABC) superfamily. Curr Med Chem Anticancer Agents 4:53–62. doi:10.2174/1568011043482160. 14754412

[35] Blair JMA, Richmond GE, Piddock LJV 2014 Multidrug efflux pumps in Gram-negative bacteria and their role in antibiotic resistance. Future Microbiol 9:1165–1177. doi:10.2217/fmb.14.66. 25405886

[36] Chung YJ, Saier J 2001 SMR-type multidrug resistance pumps. Curr Opin Drug Discov Dev 4:237–245. 11378963

[37] Fluman N, Bibi E 2009 Bacterial multidrug transport through the lens of the major facilitator superfamily. Biochim Biophys Acta 1794:738–747. doi:10.1016/j.bbapap.2008.11.020. 19103310

[38] Das D, Xu QS, Lee JY, Ankoudinova I, Huang C, Lou Y, DeGiovanni A, Kim R, Kim SH 2007 Crystal structure of the multidrug efflux transporter AcrB at 3.1 Å resolution reveals the N-terminal region with conserved amino acids. J Struct Biol 158:494–502. doi:10.1016/j.jsb.2006.12.004. 17275331 PMC2023878

[39] Takatsuka Y, Nikaido H 2006 Threonine-978 in the transmembrane segment of the multidrug efflux pump AcrB of Escherichia coli is crucial for drug transport as a probable component of the proton relay network. J Bacteriol 188:7284–7289. doi:10.1128/JB.00683-06. 17015667 PMC1636234

[40] Kumar S, Varela MF 2012 Biochemistry of bacterial multidrug efflux pumps. Int J Mol Sci 13:4484–4495. doi:10.3390/ijms13044484. 22605991 PMC3344227

[41] Li X, Elkins CA, Zgurskaya HI (ed). 2016 Efflux-mediated antimicrobial resistance in bacteria: mechanisms, regulation and clinical implications. Springer International Publishing, Cham, Switzerland.

[42] Elbrecht V, Braukmann TWA, Ivanova NV, Prosser SWJ, Hajibabaei M, Wright M, Zakharov EV, Hebert PDN, Steinke D 2019 Validation of COI metabarcoding primers for terrestrial arthropods. PeerJ 7:e7745. doi:10.7717/peerj.7745. 31608170 PMC6786254

[43] Delmont TO, Malandain C, Prestat E, Larose C, Monier JM, Simonet P, Vogel TM 2011 Metagenomic mining for microbiologists. ISME J 5:1837–1843. doi:10.1038/ismej.2011.61. 21593798 PMC3223302

[44] Lomovskaya O, Lewis K 1992 Emr, an Escherichia coli locus for multidrug resistance. Proc Natl Acad Sci U S A 89:8938–8942. doi:10.1073/pnas.89.19.8938. 1409590 PMC50039

[45] Lomovskaya O, Lewis K, Matin A 1995 EmrR is a negative regulator of the Escherichia coli multidrug resistance pump emrAB. J Bacteriol 177:2328–2334. doi:10.1128/jb.177.9.2328-2334.1995. 7730261 PMC176888

[46] Paulsen IT, Skurray RA, Tam R, Saier MH, Turner RJ, Weiner JH, Goldberg EB, Grinius LL 1996 The SMR family: a novel family of multidrug efflux proteins involved with the efflux of lipophilic drugs. Mol Microbiol 19:1167–1175. doi:10.1111/j.1365-2958.1996.tb02462.x. 8730859

[47] Nishino K, Latifi T, Groisman EA 2006 Virulence and drug resistance roles of multidrug efflux systems of Salmonella enterica serovar Typhimurium. Mol Microbiol 59:126–141. doi:10.1111/j.1365-2958.2005.04940.x. 16359323

[48] McAleese F, Petersen P, Ruzin A, Dunman PM, Murphy E, Projan SJ, Bradford PA 2005 A novel MATE family efflux pump contributes to the reduced susceptibility of laboratory-derived Staphylococcus aureus mutants to tigecycline. Antimicrob Agents Chemother 49:1865–1871. doi:10.1128/AAC.49.5.1865-1871.2005. 15855508 PMC1087644

[49] Kaatz GW, McAleese F, Seo SM 2005 Multidrug resistance in Staphylococcus aureus due to overexpression of a novel multidrug and toxin extrusion (MATE) transport protein. Antimicrob Agents Chemother 49:1857–1864. doi:10.1128/AAC.49.5.1857-1864.2005. 15855507 PMC1087643

[50] Nikaido H, Zgurskaya HI 2001 AcrAB and related multidrug efflux pumps of Escherichia coli. J Mol Microbiol Biotechnol 3:215–218. 11321576

[51] Nikaido H 1996 Multidrug efflux pumps of Gram-negative bacteria. J Bacteriol 178:5853–5859. doi:10.1128/jb.178.20.5853-5859.1996. 8830678 PMC178438

[52] Nikaido H, Basina M, Nguyen VY, Rosenberg EY 1998 Multidrug efflux pump AcrAB of Salmonella typhimurium excretes only those beta-lactam antibiotics containing lipophilic side chains. J Bacteriol 180:4686–4692. doi:10.1128/JB.180.17.4686-4692.1998. 9721312 PMC107484

[53] Masuda N, Sakagawa E, Ohya S, Gotoh N, Tsujimoto H, Nishino T 2000 Substrate specificities of MexAB-OprM, MexCD-OprJ, and MexXY-OprM efflux pumps in Pseudomonas aeruginosa. Antimicrob Agents Chemother 44:3322–3327. doi:10.1128/aac.44.12.3322-3327.2000. 11083635 PMC90200

[54] Welch A, Awah CU, Jing S, Van Veen HW, Venter H 2010 Promiscuous partnering and independent activity of MexB, the multidrug transporter protein from Pseudomonas aeruginosa. Biochem J 430:355–364. doi:10.1042/BJ20091860. 20583998

[55] Masaoka Y, Ueno Y, Morita Y, Kuroda T, Mizushima T, Tsuchiya T 2000 A two-component multidrug efflux pump, EbrAB, in Bacillus subtilis. J Bacteriol 182:2307–2310. doi:10.1128/jb.182.8.2307-2310.2000. 10735876 PMC111282

[56] Kikukawa T, Nara T, Araiso T, Miyauchi S, Kamo N 2006 Two-component bacterial multidrug transporter, EbrAB: mutations making each component solely functional. Biochim Biophys Acta 1758:673–679. doi:10.1016/j.bbamem.2006.04.004. 16750162

[57] Bay DC, Turner RJ 2012 Small multidrug resistance protein emrE reduces host pH and osmotic tolerance to metabolic quaternary cation osmoprotectants. J Bacteriol 194:5941–5948. doi:10.1128/JB.00666-12. 22942246 PMC3486072

[58] Bay DC, Rommens KL, Turner RJ 2008 Small multidrug resistance proteins: a multidrug transporter family that continues to grow. Biochim Biophys Acta 1778:1814–1838. doi:10.1016/j.bbamem.2007.08.015. 17942072

[59] Paulsen IT, Littlejohn TG, Radstrom P, Sundstrom L, Skold O, Swedberg G, Skurray RA 1993 The 3′ conserved segment of integrons contains a gene associated with multidrug resistance to antiseptics and disinfectants. Antimicrob Agents Chemother 37:761–768. doi:10.1128/aac.37.4.761. 8494372 PMC187754

[60] Cruz A, Micaelo N, Félix V, Song J-Y, Kitamura S-I, Suzuki S, Mendo S 2013 sugE: a gene involved in tributyltin (TBT) resistance of Aeromonas molluscorum Av27. J Gen Appl Microbiol 59:39–47. doi:10.2323/jgam.59.47. 23518517

[61] Chung YJ, Saier MH, Jr 2002 Overexpression of the Escherichia coli sugE gene confers resistance to a narrow range of quaternary ammonium compounds. J Bacteriol 184:2543–2545. doi:10.1128/jb.184.9.2543-2545.2002. 11948170 PMC135002

[62] Konings WN, Poelarends GJ 2002 Bacterial multidrug resistance mediated by a homologue of the human multidrug transporter P-glycoprotein. IUBMB Life 53:213–218. doi:10.1080/15216540212646. 12120998

