# Supplementary material for: Creation of Universal Primers Targeting Nonconserved, Horizontally Mobile Genes: Lessons and Considerations
Source: Appl Environ Microbiol. 2021 Jan 29;87(4):e02181-20. doi: 10.1128/AEM.02181-20 (PMC7851684; doi:10.1128/AEM.02181-20)
Supplement: Supplemental file 1 [file AEM.02181-20-s0001.pdf]

Supplementary Table 1. Complete list of all binding locations for each primer sorted into the percent homology scores

Super family	Target gene	Primer name<br>(U= upstream<br>D= downstream)	100% homology<br>(T_M °C)	90-99% homology (T_M °C)	80-89% homology<br>(T_M °C)
MFS	qacA	A.woodii qacA_U	qacA/emrB – Awo_c32030 (61.9)		
		A.woodii qacA_D	qacA/emrB – Awo_c32030 (62.0)		
		D. vulgaris qacA_U	emrB/qacA – DvMF_1099 (58.3)		
		D. vulgaris qacA_D	emrB/qacA – DvMF_1099 (55.4)		PcrA – QU35_03180 (50.3)<br><br>succinyl-CoA synthetase subsunit alpha – QU35_08905 (57.1)<br><br>Cupin – GSUB_03070 (56.2)
		P.putida qacA1/3_U	Drug resistance transporter emrB/qacA family – PP_1388 (61.8)<br><br>Putative emrB/qacA family drug resistance transporter – PP_2067 (58.3)	parB – PP_0001 (57.2)<br><br>pepP – PP_5200 (56.5)<br><br>parB-like – Tmz1t_0207 (59.0)<br><br>Acriflavin resistance protein – Tmz1t_0322 (56.2)<br><br>Conserved hypothecial protein – Tmz1t_1547 (61.2)<br><br>TviB – Tmz1t_1116 (61.9)	
		P.putida qacA1_D	Drug resistance transporter emrB/qacA family – PP_1388 (63.5)		Putative sulfate transporter – PP_0101 (60.9)<br><br>cmpX – PP_2087 (66.1)
		P.putida qacA3_D	Putative emrB/qacA family drug resistance transporter – PP_2067 (57.6)	Fiu – PP_0350 (59.8)	
		P.putida qacA4_U	emrB/qacA – PP_4951 (67.3)	Bcr/CflA – PP_3588 (64.9)	
		P.putida qacA4_D	emrB/qacA – PP_4951 (64.4)		Cytochrome C oxidase – Tmz1t_1277 (66.4)

					fimD – PP_1889 (64.7)<br><br>baeS – PP_4504 (60.1)
	emrB	A. woodii emrB_U	qacA/emrB – Awo_c03830 (54.8)		
		A. woodii emrB_D	qacA/emrB – Awo_c03830 (55.9)		
		P. putida emrB_U	emrB – PP_3548 (56.2)		
		P. putida emrB_D	emrB – PP_3548 (59.7)		mgtA – DvMF_0437 (62.0)
SMR	emrE	A. woodii emrE_U	emrE – Awo_c05160 (54.1)		Phosphoprotein phosphatase – Awo_c322070 (57.7)<br><br>Peptide ABC transporter permease – QU35_06380 (53.2)<br><br>ppiC-type peptidyl-propyl cis-trans isomerase – DvMF_3022 (58.5)<br><br>yfeH – PP_3247 (61.0)
		A. woodii emrE_D	emrE – Awo_c05160 (54.4)		
		B. subtilis emrE_U	emrE – QU35_06845 (54.2)		Non-coding region – GSUB:834835 (50.1)
		B. subtilis emrE_D	QU35_06845 (54.4)		tlyA – Awo_c13160 (52.7)<br><br>polC2 – Awo_c25450 (56.7)
		P. putida emrE_U	emrE – PP_4930 (62.9)	betA – PP_3383 (67.6)	
		P. putida emrE_D	emrE – PP_4930 (64.1)		YaaH (sporulation protein) – QU35_03275 (60.3)<br><br>D-tyrosyl-tRNA(Tyr) deacylase – DvMF_0255 (69.3)<br><br>transcriptional regulator, LuxR family – DvMF_0929 (69.9)<br><br>translation elongation factor Tu – DvMF_1447 (67.9)<br><br>Universal stress protein family – PP_1269 (64.9)<br><br>Transcriptional regulator, AraC family – PP_3526 (62.3)

					Conserved hypothetical protein – Tmz1t_0375 (64.9)<br><br>Conserved hypothetical protein – Tmz1t_0977 (62.9)<br><br>DNA polymerase I – Tmz1t_3813 (64.6)
	qacE	B. subtilis qacE1_U	qacE – QU35_18255 (54.3)		
		B. subtilis qacE1_D	qacE – QU35_18255 (53.9)		
		B. subtilis qacE2_U	emrE – QU35_18720 (54.3)		glgP1 – Awo_c03450 (54.6)<br><br>Glucose-6-phosphate dehydrogenase – QU35_13040 (52.1)<br><br>ABC transporter permease – QU35_21320 (51.6)
		B. subtilis qacE2_D	emrE – QU35_18720 (54.3)		
	sugE	G. subterraneus sugE_U	eamA – GSUB_08270 (52.2)		
		G. subterraneus sugE_D	eamA – GSUB_08270 (52.4)		Ribose 5-phosphate isomerase – QU35_19985 (52.8)<br><br>ATPase AAA – GSUB_10105 (54.3)
		P. putida sugE_U	sugE – PP_1701 (54.6)		
		P. putida sugE_D	sugE – PP_1701 (53.7)		tkrH1 – Awo_c05550 (50.5)<br><br>hypothetical protein – QU35_15730 (54.0)<br><br>iron ABC transporter permease – QU35_18115 (58.1)<br><br>FtsH – GSUB_01090 (56.1)
	ebrA	B. subtilis ebrA_U	ebrA – QU35_09520 (58.0)		
		B. subtilis ebrA_D	ebrA – QU35_09520 (58.0)		
ABC	lmrA	B. subtilis lmrA_U	multidrug MFS transporter – QU35_01610 (53.7)		
		B. subtilis lmrA_D	multidrug MFS transporter – QU35_01610 (53.7)		

MATE	norM	D. vulgaris norM_U	norM – DvMF_3111 (66.8)	ycdD-like – PP_2694 (68.6)<br><br>hemN_C – Tmz1t_4026 (64.1)	
		D. vulgaris norM_D	norM – DvMF_3111 (64.4)	hisJ – DvMF_0350 (68.1)<br><br>hypothetical protein – DvMF_1904 (66.7)<br><br>rpmB – Tmz1t_1252 (61.7)	
		P. putida norM_U	norM – PP_5262 (65.9)		
		P. putida norM_D	norM – PP_5262 (59.6)		
		T. aromatica norM_U	norM – Tmz1t_3585 (65.6)	Ethanolamine ammonia lyase large subunit – DvMF_1253 (65.6)<br><br>NAD synthase – GSUB_13065 (69.0)<br><br>MFS transporter – PP_2935 (64.6)<br><br>dctM – Tmz1t_0544 (63.8)<br><br>yfdV – Tmz1t_0790 (69.0)	
		T. aromatica norM_D	norM – Tmz1t_3585 (69.0)		
	matE	A. woodii matE1_U	matE5 – Awo_c29700 (53.4)		
		A. woodii matE1_D	matE5 – Awo_c29700 (53.3)		
		A. woodii matE2_U	matE6 – Awo_c32280 (53.4)		
		A. woodii matE2_D	matE6 – Awo_c32280 (53.3)		
	mepA	A. woodii mepA_U	matE8 – Awo_c34640 (53.3)		
		A. woodii mepA_D	matE8 – Awo_c34640 (53.5)		
RND	acrB	G. subterraneus acrB1_U	acrB – GSUB_09845 (60.1)		
		G. subterraneus acrB1_D	acrB – GSUB_09845 (60.2)		ABC-type transporter ATP binding protein – Awo_c08500 (61.3)<br><br>Hypothetical protein – GSUB_09950 (62.9)<br><br>Putative periplasmic aliphatic sulfonate binding protein – PP_3228 (64.5)

		G. subterraneus acrB2_U	acrB – GSUB_10570 (60.9)		phnJ – GSUB_11850 (59.2)
		G. subterraneus acrB2_D	acrB – GSUB_10570 (60.3)		HAE1 – DvMF_0036 (66.0)
		G. subterraneus acrB3_U	acrB – GSUB_14010 (60.2)		ymfN – PP_1563 (60.4)<br><br>sucA – PP_4189 (64.4)<br><br>hisKA – Tmz1t_2260 (59.0)<br><br>Conserved protein of unknown function – PP_4200 (65.9)<br><br>Conserved protein of unknown function – PP_5159 (66.1)
		G. subterraneus acrB3_D	acrB – GSUB_14010 (60.5)		MFS_1 – Tmz1t_1609 (65.0)
		T. aromatica acrB1_U	acrB – Tmz1t_0322 (60.5)	acrB – Tmz1t_2073 (63.4)	
		T. aromatica acrB1_D	acrB – Tmz1t_2073 (60.2)<br><br>acrB – Tmz1t_0322 (60.2)	Wzy family polymerase, exosortase system – Tmz1t_3268 (64.8)	Sulfate ABC transporter – Tmz1t_0628 (64.2)<br><br>Mfd – Tmz1t_2220 (58.2)<br><br>envC – Tmz1t_1545 (70.3)<br><br>NADH dehydrogenase (quinone) – Tmz1t_2445 (63.8)<br><br>hsdM1 – Awo_c04420 (56.9)<br><br>ABC-type uncharacterized transport system – Tmz1t_1950 (67.4)<br><br>Citrate transporter – QU35_21100 (60.4)<br><br>mreC – PP_0934 (60.9)<br><br>Hypothetical protein – DvMF_2951 (63.6)<br><br>Putative zinc uptake regulation protein – PP_0119 (58.5)<br><br>ridA – PP_5303 (55.3)<br><br>mexB – PP_3456 (65.8)

		T. aromatica acrB3/5_U	acrB – Tmz1t_2073 (61.5)<br><br>acrB – Tmz1t_3302 (59.2)	acrB – Tmz1t_0322 (60.5)<br><br>moeA – DvMF_1797 (66.5)<br><br>Translation elongation factor Tu – DvMF_1447 (61.5)<br><br>thrS – DvMF_0984 (61.5)<br><br>msbA – Tmz1t_3812 (58.9)<br><br>Sensor histidine kinase/response regulator – PP_1875 (60.9)<br><br>Conserved exported protein of unknown function – PP_1230 (61.4)<br><br>Alpha/beta hydrolase fold protein – Tmz1t_1678 (59.1)	
		T. aromatica acrB3_D	acrB – Tmz1t_2073 (64.3)	acrB – Tmz1t_0322 (61.0)	
		T. aromatica acrB5_D	acrB – Tmz1t_3302 (58.9)		
		T. aromatica acrB4/6/7_U	acrB – Tmz1t_3460 (61.9)<br><br>acrB – Tmz1t_2714 (64.1)<br><br>acrB – Tmz1t_3537 (64.1)	Putative Ca^{2+}/H^+ antiporter – Tmz1t_3536 (60.5)<br><br>Hypothetical protein – Tmz1t_0560 (63.2)<br><br>fsr-I – PP_0701 (61.2)<br><br>Binding-protein-dependent transport systems inner membrane – Tmz1t_2806 (64.1)<br><br>NADH/Ubiquinone/plastoquinone (complex I) – Tmz1t_2443 (66.4)	
		T. aromatica acrB4_D	acrB – Tmz1t_2714 (61.5)	Putative metabolite efflux pump – PP_4568 (63.4)	ABC transporter related – DvMF_2156 (61.6)<br><br>Translation initiation factor – DvMF_2235 (61.5)

					arsB_nhaD permease – Tmzt1_0432 (68.1)
		T. aromatica acrB6_D	acrB – Tmz1t_3460 (68.6)		pad – Tmz1t_1510 (65.3)<br><br>dapE – Tmz1t_2196 (64.5)<br><br>tolQ – Tmz1t_0785 (61.7)<br><br>ligA – Tmz1t_2190 (73.2)<br><br>cls – DvMF_1528 (66.9)<br><br>Phosphoenolpyruvate synthase – Tmz1t_2655 (64.1)<br><br>Glycosyl transferase – PP_3256 (63.6)
		T. aromatica acrB7_D	acrB – Tmz1t_3537 (67.0)	arcA – PP_1001 (69.6)<br><br>lysE – PP_0198 (65.7)<br><br>GAF sensor signal transduction histidine kinase – Tmz1t_2631 (67.0)	rfaB – DvMF_1833 (73.8)<br><br>mob – Tmz1t_2526 (66.4)<br><br>htpG – PP_4179 (64.0)<br><br>endonuclease/exonucle ase/phosphatase – Tmz1t_3642 (61.9)
		P. putida acrB_D	acrB – PP_2065 (63.0)	HAE1 – Tmz1t_0505 (60.1)<br><br>acrB – Tmz1t_1816 (60.1)	mexF – PP_3426 (60.2)<br><br>Methenyltetrahydrofol ate cyclohydrolase – Tmz1t_3190 (67.0)<br><br>Non-coding region – PP:2477867 (63.8)<br><br>Putative RND transporter – PP_0906 (60.2)<br><br>mexD – PP_2818 (60.2)
		T. aromatica acrB2_U	acrB – Tmz1t_1816 (62.2)		
	acrB/ mexD	P. putida acrB/mexD_U	acrB – PP_2065 (62.8)<br><br>mexD – PP_2818 (60.1)	HAE1 – DvMF_0036 (61.9)<br><br>HAE1 – GSUB_04250 (57.7)<br><br>mdtB – PP_3584 (59.1)	

				RNA methyltransferase – DvMF_1155 (57.4)<br><br>HAE1 – Tmz1t_0505 (64.1)<br><br>ttgB – PP_1385 (61.9)<br><br>mexF – PP_3426 (61.9)<br><br>mexB – PP_3456 (60.5)	
		T. aromatica acrB2/ P. putida mexD_D	mexD – PP_2818 (60.2)<br><br>acrB – Tmz1t_1816 (60.1)<br><br>Putative RND transporter – PP_0906 (60.2)<br><br>mexF – PP_3426 (60.2)<br><br>mexB – PP_3456 (58.1)<br><br>HAE1 – Tmz1t_0505 (60.1)	ttgB – PP_1385 (57.0)<br><br>acrB – PP_2065 (63.0)	nudL – PP_1453 (56.2)<br><br>hypothetical protein – QU35_16560 (55.8)<br><br>Histidine kinase – GSUB_12130 (58.0)<br><br>hypothetical protein – Awo_c23040 (53.9)<br><br>4Fe-4S ferredoxin iron-sulfur binding domain protein – DvMF_0593 (60.2)<br><br>Hypothetical protein – Tmz1t_0229 (68.7)<br><br>CoA-binding domain protein – Tmz1t_2041 (68.5)<br><br>Ketol-acid reductisomerase – Tmz1t_2775 (64.1)<br><br>acrB – Tmz1t_3302 (58.4)
	HAE1	D. vulgaris HAE1_U	HAE1 – DvMF_0036 (60.7)	HAE1 – Tmz1t_0505 (60.7)	
		D. vulgaris HAE1_D	HAE1 – DvMF_0036 (59.0)	acrB – Tmz1t_3302 (61.4)<br><br>acrB – Tmz1t_3460 (59.1)<br><br>ttg – PP_1385 (61.3)<br><br>acrB – PP_3302 (57.3)<br><br>mexF – PP_3426 (59.0)	

				histone deacetylase – PP_4764 (61.2)<br><br>acrB – GSUB_09845 (59.0)<br><br>acrB – DvMF_1515 (61.3)<br><br>PTS – DvMF_2584 (61.6)	
		D. vulgaris HAE2_U	HAE1 – DvMF_2163 (54.8)		
		D. vulgaris HAE2_D	HAE1 – DvMF_2163 (56.8)	HAE1 – GSUB_04250 (52.0)<br><br>metQ – PP_0112 (60.9)<br><br>methyl transferase small – Tmz1t_1562 (60.9)	
		G. subterraneus HAE1_U	HAE1 – GSUB_04250 (52.1)	PP_2065 (54.6)	
		G. subterraneus HAE1_D	HAE1 – GSUB_04250 (52.0)	HAE1 – DvMF_2163 (56.8)	
		G. subterraneus HAE2_U	HAE1 – GSUB_06495 (56.4)	HAE1 – Tmz1t_0505 (60.7)	
		G. subterraneus HAE2_D	HAE1 – GSUB_06495 (59.3)	HAE1 – GSUB_04250 (57.4)<br><br>Amidophosphoribosyl transferase – QU35_03740 (52.9)<br><br>HAE1 – DvMF_2163 (61.6)<br><br>mexD – PP_2818 (61.6)<br><br>mdtC – PP_3583 (59.3)<br><br>acrB – Tmz1t_0322 (62.0)<br><br>HAE1 – Tmz1t_0505 (61.6)<br><br>acrB – Tmz1t_2073 (62.0)	
		T. aromatica HAE1_U	Tmz1t_	HAE1 – DvMF_0036 (60.7)	

				HAE1 – GSUB_06495 (56.4)<br><br>mexB – PP_3456 (60.7)	
		T. aromatica HAE1_D	HAE1 – Tmz1t_0505 (61.6)<br><br>mexD – PP_2818 (61.6)<br><br>HAE1 – DvMF_2163 (61.6)	HAE1 – GSUB_04250 (57.4)<br><br>GSUB_06495 (59.3)<br><br>acrB – Tmz1t_1816 (59.4)<br><br>acrB – Tmz1t_2073 (62.0)<br><br>acrB – Tmz1t_2714 (59.8)<br><br>amidophosphoribosyl transferase – Tmz1t_3056 (58.4)<br><br>acrB – Tmz1t_0322 (62.0)<br><br>mexB – PP_3456 (59.4)<br><br>putative RND transporter – PP_0906 (57.4)<br><br>purF – PP_2000 (58.4)<br><br>acrB – PP_2065 (57.4)<br><br>mdtC – PP_3583 (59.3)<br><br>Glutamyl-tRNA synthetase – DvMF_0996 (61.6)<br><br>Pyruvate flavodoxin/ferredoxin oxidoreductase domain protein – DvMF_2055 (64.6)	

Supplementary Table 2. Complete list of percent identity scores for 16S rRNA genes identified in the genomes of the six (6) organisms used in this study (MSA length 1595)

Species	16S gene (nucleotide position)	Number of mismatches	Percent identity score
Acetobacterium woodii	rrsA (88307-89819)	264	83.45
	rrsB (389170-390683)	265	83.39
	rrsC (1098747-1100259)	265	83.39
	rrsD (2819873-2821385)	267	83.26
	rrsE (3344665-3346177)	267	83.26
Bacillus subtilis	QU35_00040 (9810-11365)	148	90.72
	QU35_00150 (30279-31833)	148	90.72
	QU35_00485 (90536-92090)	149	90.66
	QU35_00545 (96392-97946)	149	90.66
	QU35_00950 (160893-162447)	148	90.72
	QU35_00995 (166503-168057)	148	90.72
	QU35_01010 (171505-173058)	149	90.66
	QU35_03385 (635443-636998)	147	90.78
	QU35_04910 (946706-948261)	148	90.72
	QU35_16875 (3177098-3178653)	150	90.60
Desulfovibrio vulgaris	DvMF_R0027 (1108180-1109715)	316	80.19
	DvMF_R0034 (1281729-1283264)	316	80.19
	DvMF_R0056 (2773365-2774900)	316	80.19
Geoalkalibacter subterraneus	GSUB_12160 (2611130-2612695)	249	84.39
	GSUB_12185 (2616805-2624045)	249	84.39
	GSUB_12210 (2622480-2624045)	248	84.45
Pseudomonas putida	PP_16SA (171389-172910)	279	82.51
	PP_16SB (176819-178340)	279	82.51
	PP_16SC (524949-526470)	280	82.45
	PP_16SD (697824-699345)	278	82.57
	PP_16SE (1325503-1327024)	280	82.45
	PP_16SF (2548690-2550211)	279	82.51
	PP_16SG (5311160-5312681)	279	82.51
Thauera aromatica	Tmz1T_R0002 (454429-455955)	336	78.93
	Tmz1T_R0016 (1779410-1780936)	336	78.93
	Tmz1T_R0059 (3499877-3501403)	336	78.93
	Tmz1T_R0074 (4024356-4025882)	336	78.93
Average		244 ± 69	84.71 ± 4.32

Supplementary Table 3. Complete list of percent identity scores for the *qacA/emrB* genes as a member of the major facilitator superfamily (MFS) superfamily identified in the genomes of the six (6) organisms used in this study (MSA length 1630)

Species	*qacA/emrB* gene (nucleotide position)	Number of mismatches	Percent identity score
Acetobacterium woodii	Awo_c32030 (3670888-3672336)	822	49.57
Desulfovibrio vulgaris	DvMF_1099 (1345563-1347122)	554	66.01
Pseudomonas putida	PP_1388 (1583562-1585013)	725	55.52
	PP_1975 (2237033-2238583)	588	63.93
	PP_2067 (2351750-2353264)	623	61.78
	PP_4951 (5640338-5641765)	783	51.96
Average		683 ± 100	58.13 ± 6.15

Supplementary Table 4. Complete list of percent identity scores for the *emrE* genes as a member of the small multidrug resistance (SMR) superfamily identified in the genomes of the six (6) organisms used in this study (MSA length 1137)

Species	*emrE* gene (nucleotide position)	Number of mismatches	Percent identity score
Acetobacterium woodii	Awo_c05160 (594880-595926)	360	68.34
Bacillus subtilis	QU35_06845 (296630-1297604)	439	61.39
Pseudomonas putida	PP_4930 (5610586-5610918)	907	20.23
Average		569 ± 241	49.99 ± 21.23

Supplementary Table 5. Complete list of percent identity scores for the *norM* genes as a member of the multidrug and toxic (compound) extrusion (MATE) superfamily identified in the genomes of the six (6) organisms used in this study (MSA length 1521)

Species	*norM* gene (nucleotide position)	Number of mismatches	Percent identity score
Desulfovibrio vulgaris	DvMF_3111 (3923820-3925289)	509	66.54
Pseudomonas putida	PP_5262 (6007715-6009103)	499	67.19
Thauera aromatica	Tmz1T_3585 (3930672-3932090)	469	69.17
Average		492 ± 17.00	67.63 ± 1.11

Supplementary Table 6. Complete list of percent identity scores for the *acrB* gene as a member of the resistance-nodulation-cell division (RND) superfamily identified in the genomes of the six (6) organisms used in this study (MSA length 3990)

Species	*acrB* gene (nucleotide position)	Number of mismatches	Percent identity score
Geoalkalibacter subterraneus	GSUB_09845 (2133932-2137006)	2240	43.86
	GSUB_10570 (2277540-2277893)*	3780	5.26
	GSUB_12010 (2577785-2578063)*	3821	4.24
Pseudomonas putida	PP_2065 (2347794-2350877)	2114	47.02
Thauera aromatica	Tmz1t_0322 (365083-368391)	1536	61.50
	Tmz1t_1816 (2037646-2040717)	1973	50.55
	Tmz1t_2073 (2345734-2349042)	1530	61.65
	Tmz1t_2714 (2942913-2945966)	1978	50.43
	Tmz1t_3302 (3617336-3620626)	1744	56.29
	Tmz1t_3460 (3782412-3785633)	1914	52.03
	Tmz1t_3537 (3875412-3878489)	1954	51.03
Average (average minus short gene annotations)		2234 ± 766 (1887 ± 228)	43.99 ± 19.21 (52.71 ± 5.71)

* Significantly shorter gene sequences

Supplementary Table 7. Complete list of all binding locations for each *leuC* primer sorted into the percent homology scores

Target gene	Primer name (U= upstream D= downstream)	100% homology (T_M °C)	90-99% homology (T_M °C)	80-89% homology (T_M °C)
leuC	MSA1_degen_U	leuC1 – Awo_c02850 (55.6)<br><br>leuC2 – Awo_c13960 (57.1)<br><br>leuC – DvMF_1792 (65.6)<br><br>leuC – GSUB_10900 (62.3)	Von Willebrand factor type A – DvMF_1495 (59.1)<br><br>leuC – PP_1985 (65.3)	Peptide-synthetase – QU35_10100 (53.6)<br><br>Non-coding region – QU35:2750774 (53.4)<br><br>Non-coding region – DvMF:1089734 (62.3)<br><br>CstA – DvMF_1226 (70.4)<br><br>CstA – DvMF_1229 (70.4)<br><br>Sulfate transporter – DvMF_1575 (63.3)

				CRISPR recognition tool – DvMF:2444693-2448151 (64.6)<br><br>Permease – DvMF_2194 (62.7)<br><br>Ferredoxin-dependent glutamate synthase – DvMF_2388 (71.1)<br><br>Glycosyl transferase, group 2 – DvMF_2395 (65.3)<br><br>Molybdopterin oxidoreductase – DvMF_2470 (69.2)<br><br>hypothetical protein – GSUB_05170 (60.0)<br><br>dgcA – PP_0310 (58.9)<br><br>conserved protein of unknown function – PP_0784 (62.4)<br><br>non-coding region – PP:1389271 (69.7)<br><br>leuC – PP_1985 (65.2)<br><br>Iron-sulfur cluster-binding protein – PP_3543 (65.2)<br><br>leuC – Tmz1t_3071 (68.2)<br><br>Non-coding region – Tmz1t:3513414 (65.8)<br><br>Non-coding region – Tmz1t:4255927 (59.5)
	MSA1_degen_D	leuC1 – Awo_c02850 (55.1)<br><br>leuC2 – Awo_c13960 (58.9)<br><br>leuC – DvMF_1792 (61.7)<br><br>leuC – Tmz1t_3071 (61.8)	oppF4 – Awo_c00460 (57.0)<br><br>alpha/beta superfamily hydrolase – Awo_c31750 (60.8)<br><br>adenine deaminase – QU35_08085 (56.3)<br><br>transporter – QU35_12980 (57.8)<br><br>leuC – GSUB_10900 (59.9)<br><br>leuC – PP_1985 (57.2)<br><br>extracellular ligand-binding receptor – Tmz1t_1517 (64.4)	

	LeuC_Awoo1_U	leuC1 – Awo_c02850 (55.6)	leuC – GSUB_10900 (62.3)	
	LeuC_Awoo1_D	leuC1 – Awo_c02850 (55.1)	leuC – GSUB_10900 (59.9)	
	LeuC_Awoo2_U	leuC2 – Awo_c13960 (57.1)		
	LeuC_Awoo2_D	leuC2 – Awo_c13960 (58.9)	leuC – Tmz1t_3071 (61.8)	
	LeuC_Dvulg_U	leuC – DvMF_1792 (65.6)		
	LeuC_Dvulg_D	leuC – DvMF_1792 (61.7)	leuC – GSUB_10900 (59.9)<br><br>leuC – Tmz1t_3071 (61.8)	
	LeuC_Gsub_U	leuC – Gsub_10900 (62.3)		leuC1 – Awo_c02850 (55.6)
	LeuC_Gsub_D	leuC – Gsub_10900 (59.9)	leuC1 – Awo_c02850 (55.1)<br><br>leuC – DvMF_1792 (61.7)<br><br>leuC – PP_1985 (57.2)	oppF4 – Awo_c00460 (57.0)<br><br>leuC2 – Awo_c13960 (58.9)<br><br>alpha/beta superfamily hydrolase – Awo_c31750 (60.8)<br><br>membrane protein – QU35_05335 (58.0)<br><br>2-isopropylmalate synthase – DvMF_1791 (54.0)<br><br>L-lactate transport – DvMF_2129 (56.9)<br><br>prfC – PP_0872 (62.2)<br><br>cynX – PP_0970 (62.8)<br><br>stcD – PP_4753 (64.6)<br><br>leuC – Tmz1t_3071 (61.8)<br><br>extracellular ligand-binding receptor – Tmz1t_1517 (64.4)

				Enoyl-CoA hydratase/isomerase – Tmz1t_2492 (62.2)
	MSA2_degen_U	leuC – QU35_15365 (50.6)<br><br>leuC – PP_1985 (58.4)<br><br>leuC – Tmz1t_3071 (58.5)	Aconitate hydratase – DvMF_3021 (54.8)<br><br>omega amino acid-pyruvate aminotransferase – PP_0596 (61.7)	recF – QU35_00020 (53.1)<br><br>hypothetical protein – GSUB_04725 (57.8)<br><br>redoxin domain protein – Tmz1t_0091 (58.4)
	MSA2_degen_D	leuC – QU35_15365 (50.6)<br><br>leuC – DvMF_1792 (56.2)<br><br>leuC – PP_1985 (56.2)<br><br>leuC – Tmz1t_3071 (56.2)		
	LeuC_Bsub_U	leuC – QU35_15365 (55.4)	leuC – PP_1985 (58.4)<br><br>leuC – Tmz1t_3071 (58.5)	recF – QU35_00020 (53.1)<br><br>omega-amino acid- pyruvate aminotransferase – PP_0595 (61.7)
	LeuC_Bsub_D	leuC – QU35_15365 (50.6)		
	LeuC_Pputi_U	leuC – PP_1985 (58.4)	leuC – QU35_15365 (55.4)<br><br>aconitase hydratase – DvMF_3021 (54.8)<br><br>omega-amino acid-pyruvate aminotransferase – PP_0596 (61.7)<br><br>leuC – Tmz1t_3071 (58.5)	hypothetical protein – GSUB_04725 (57.8)
	LeuC_Pputi_D	leuC – DvMF_1792 (56.2)<br><br>leuC – PP_1985 (56.2)<br><br>leuC – Tmz1t_3071 (56.2)		

	LeuC_Taro_U	leuC – Tmz1t_3071 (58.5)	leuC – QU35_15365 (55.4)<br><br>leuC – PP_1985 (58.4)	Omega-amino acid-pyruvate aminotransferase – PP_0596 (61.7)<br><br>Redoxin domain protein – Tmz1t_0091 (58.4)
	LeuC_Taro_D	leuC – DvMF_1792 (56.2)<br><br>leuC – Tmz1t_3071 (56.2)		

Supplementary Figure 1. Gel electrophoresis in 1.5% agarose for 45 minutes at 100 volts and stained using Gel Red dye of PCR products using universal 16S primers tested against DNA extracted from pure cultures and fresh water surface samples with and without biocide treatments.

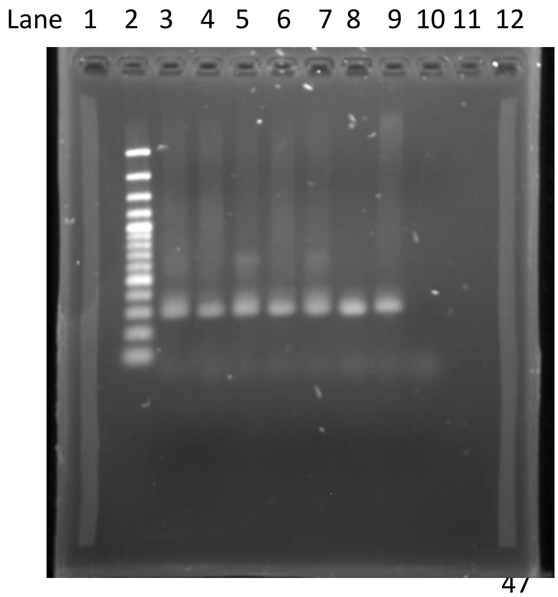

Lane	Sample
1	Empty
2	100 bp ladder
3	Universal 16S primers against untreated surface water template
4	Universal 16S primers against surface water treated with 250 parts per million (ppm) bronopol template
5	Universal 16S primers against surface water treated with 250 ppm 15% glutaraldehyde template
6	Universal 16S primers against surface water treated with 250 ppm DBNPA template
7	Universal 16S primers against surface water treated with 250 ppm 14% glutaraldehyde and 3% quaternary ammonium compound template
8	Universal 16S primers against *P. putida* template
9	Universal 16S primers against *T. aromatica* template
10	Universal 16S primers against non-template control (NTC)
11	Empty
12	Empty

Supplementary Figure 2. Gel electrophoresis in 1.5% agarose for 45 minutes at 100 volts and stained using Gel Red dye of PCR products using primers targeting *qacA1* from *P. putida* tested against DNA extracted from pure cultures and fresh water surface samples with and without biocide treatments.

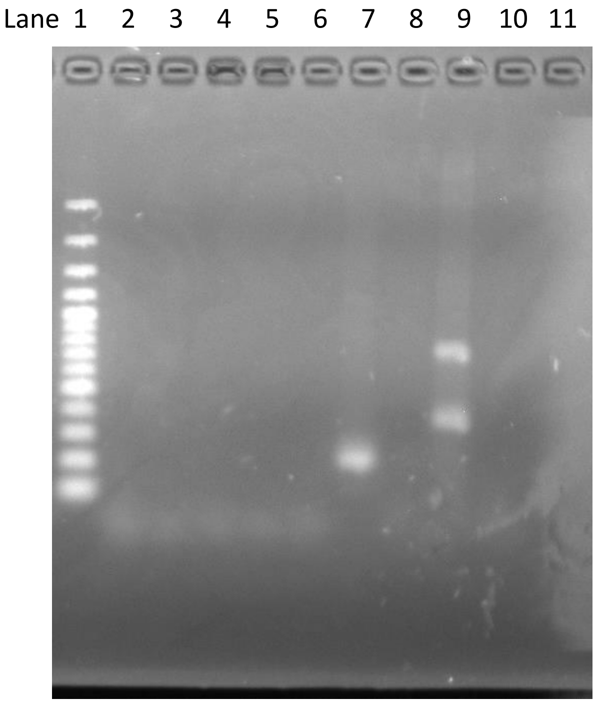

Lane	Sample
1	100 bp ladder
2	*P. puti* qacA1/3_U and *P. puti* qacA1_D primers against untreated surface water template
3	*P. puti* qacA1/3_U and *P. puti* qacA1_D primers against surface water treated with 250 ppm bronopol template
4	*P. puti* qacA1/3_U and *P. puti* qacA1_D primers against surface water treated with 250 ppm 15% glutaraldehyde template
5	*P. puti* qacA1/3_U and *P. puti* qacA1_D primers against surface water treated with 250 ppm DBNPA template
6	*P. puti* qacA1/3_U and *P. puti* qacA1_D primers against surface water treated with 250 ppm 14% glutaraldehyde and 3% quaternary ammonium compound template
7	*P. puti* qacA1/3_U and *P. puti* qacA1_D primers against *P. putida* template
8	*P. puti* qacA1/3_U and *P. puti* qacA1_D primers against NTC
9	*P. puti* qacA1/3_U and *P. puti* qacA1_D primers against *T. aromatica* template
10	*P. puti* qacA1/3_U and *P. puti* qacA1_D primers against NTC
11	Empty

Note: Two NTC lanes used as PCR were performed on separate days

Supplementary Figure 3. Gel electrophoresis in 1.5% agarose for 45 minutes at 100 volts and stained using Gel Red dye of PCR products using select MDREP primers targeting *mexD* from *P. putida* and *acrB2* from *T. aromatica* tested against DNA extracted from pure cultures and fresh water surface samples with and without biocide treatments.

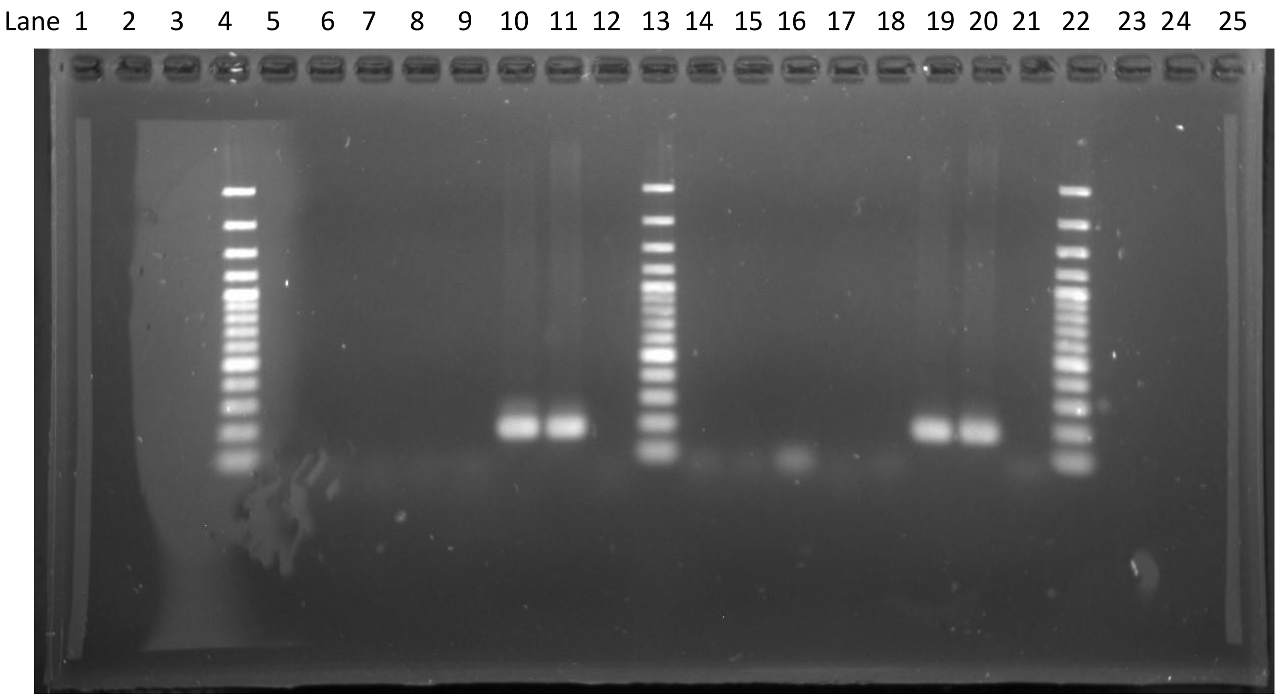

Lane	Sample
1	Empty
2	Empty
3	Empty
4	100 bp ladder
5	*P. puti* acrB/mexD_U and *T. aro* acrB2/*P. puti* mexD_D primers against untreated surface water template
6	*P. puti* acrB/mexD_U and *T. aro* acrB2/*P. puti* mexD_D primers against surface water treated with 250 parts per million (ppm) bronopol template
7	*P. puti* acrB/mexD_U and *T. aro* acrB2/*P. puti* mexD_D primers against surface water treated with 250 ppm 15% glutaraldehyde template
8	*P. puti* acrB/mexD_U and *T. aro* acrB2/*P. puti* mexD_D primers against surface water treated with 250 ppm DBNPA template
9	*P. puti* acrB/mexD_U and *T. aro* acrB2/*P. puti* mexD_D primers against surface water treated with 250 ppm 14% glutaraldehyde and 3% quaternary ammonium compound template
10	*P. puti* acrB/mexD_U and *T. aro* acrB2/*P. puti* mexD_D primers against *P. putida* template
11	*P. puti* acrB/mexD_U and *T. aro* acrB2/*P. puti* mexD_D primers against *T. aromatica* template
12	*P. puti* acrB/mexD_U and *T. aro* acrB2/*P. puti* mexD_D primers against NTC
13	100 bp ladder
14	*T.aro* acrB2_U and *T.aro* acrB2/*T.aro* mexD_D primers against untreated surface water template
15	*T.aro* acrB2_U and *T.aro* acrB2/*T.aro* mexD_D primers against surface water treated with 250 ppm bronopol template
16	*T.aro* acrB2_U and *T.aro* acrB2/*T.aro* mexD_D primers against surface water treated with 250 ppm 15% glutaraldehyde template
17	*T.aro* acrB2_U and *T.aro* acrB2/*T.aro* mexD_D primers against surface water treated with 250 ppm DBNPA template
18	*T.aro* acrB2_U and *T.aro* acrB2/*T.aro* mexD_D primers against surface water treated with 250 ppm 14% glutaraldehyde and 3% quaternary ammonium compound template
19	*T.aro* acrB2_U and *T.aro* acrB2/*T.aro* mexD_D primers against *P. putida* template
20	*T.aro* acrB2_U and *T.aro* acrB2/*T.aro* mexD_D primers against *T. aromatica* template
21	*T.aro* acrB2_U and *T.aro* acrB2/*T.aro* mexD_D primers against NTC
22	100 bp ladder
23	Empty
24	Empty
25	Empty

Supplementary Figure 4. Gel electrophoresis in 1.5% agarose for 45 minutes at 100 volts and stained using Gel Red dye of PCR products using select MDREP primers targeting *acrB* from *P. putida* tested against DNA extracted from pure cultures and fresh water surface samples with and without biocide treatments using different annealing temperatures.

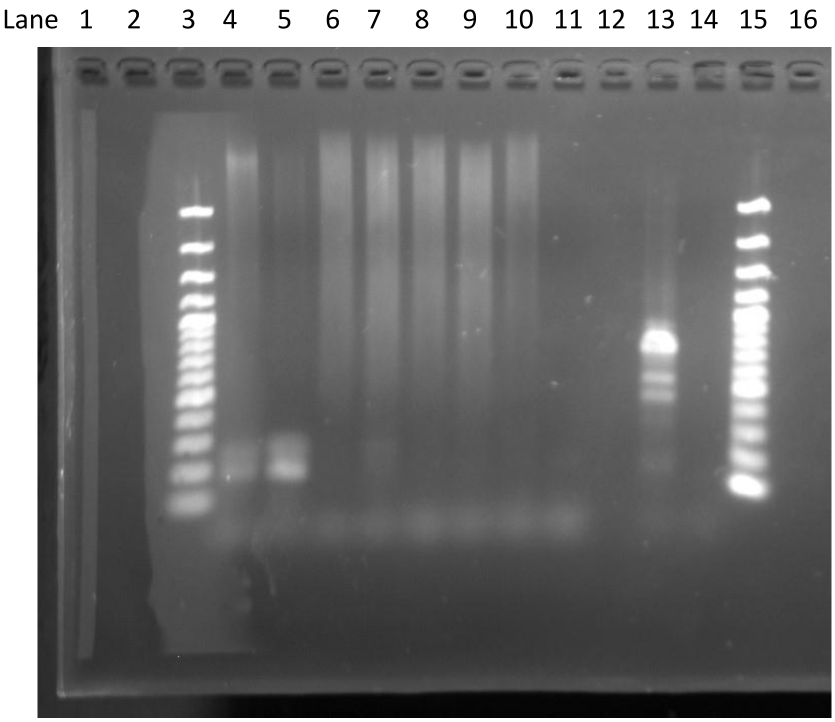

Lane	Sample
1	Empty
2	Empty
3	100 bp ladder
4	*P. puti* acrB/mexD_U and *P. puti* acrB_D primers against *T. aromatica* template (63 °C anneal)
5	*P. puti* acrB/mexD_U and *P. puti* acrB_D primers against *P. putida* template (63 °C anneal)
6	*P. puti* acrB/mexD_U and *P. puti* acrB_D primers against untreated surface water template (63 °C anneal)
7	*P. puti* acrB/mexD_U and *P. puti* acrB_D primers against surface water treated with 250 parts per million (ppm) bronopol template (63 °C anneal)
8	*P. puti* acrB/mexD_U and *P. puti* acrB_D primers against surface water treated with 250 ppm 15% glutaraldehyde template (63 °C anneal)
9	*P. puti* acrB/mexD_U and *P. puti* acrB_D primers against surface water treated with 250 ppm DBNPA template (63 °C anneal)
10	*P. puti* acrB/mexD_U and *P. puti* acrB_D primers against surface water treated with 250 ppm 14% glutaraldehyde and 3% quaternary ammonium compound template (63 °C anneal)
11	*P. puti* acrB/mexD_U and *P. puti* acrB_D primers against non-template control (NTC) (63 °C anneal)
12	Empty
13	*P. puti* acrB/mexD_U and *P. puti* acrB_D primers against *P. putida* template (58 °C anneal)
14	*P. puti* acrB/mexD_U and *P. puti* acrB_D primers against NTC (58 °C anneal)
15	100 bp ladder
16	Empty

Supplementary Figure 5. Gel electrophoresis in 1.5% agarose for 45 minutes at 100 volts and stained using Gel Red dye of PCR products using select MDREP primers targeting *acrB* from *P. putida* and *acrB2* from *T. aromatica* tested against DNA extracted from pure cultures and fresh water surface samples with and without biocide treatments, spiked with 1% DNA template from *P. putida* and *T. aromatica*.

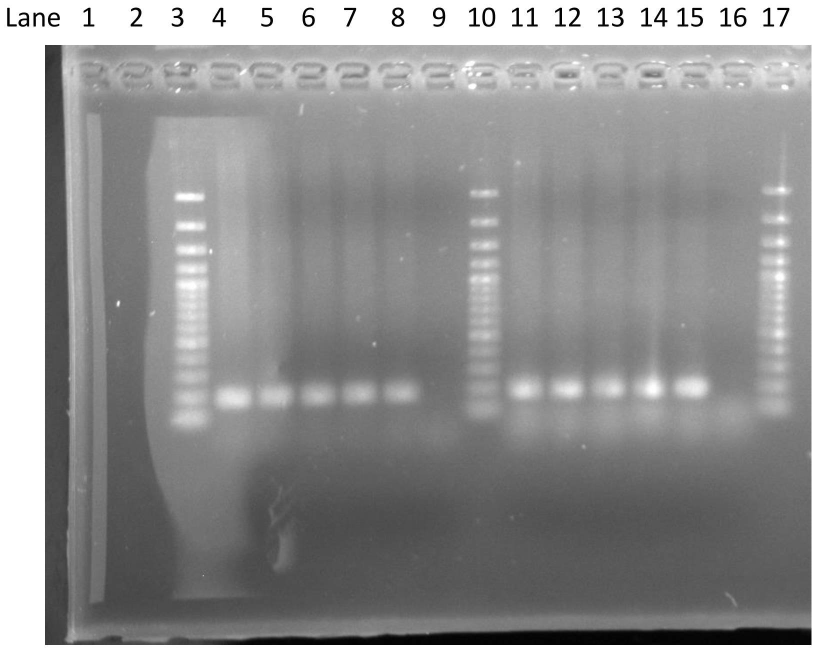

Lane	Sample
1	Empty
2	Empty
3	100 bp ladder
4	*P. puti* acrB/mexD_U and *P. puti* acrB_D primers against untreated surface water template with 1% *P. putida* genomic DNA and 1% *T. aromatica* genomic DNA
5	*P. puti* acrB/mexD_U and *P. puti* acrB_D primers against surface water treated with 250 parts per million (ppm) bronopol template with 1% *P. putida* genomic DNA and 1% *T. aromatica* genomic DNA
6	*P. puti* acrB/mexD_U and *P. puti* acrB_D primers against surface water treated with 250 ppm 15% glutaraldehyde template with 1% *P. putida* genomic DNA and 1% *T. aromatica* genomic DNA
7	*P. puti* acrB/mexD_U and *P. puti* acrB_D primers against surface water treated with 250 ppm DBNPA template with 1% *P. putida* genomic DNA and 1% *T. aromatica* genomic DNA
8	*P. puti* acrB/mexD_U and *P. puti* acrB_D primers against surface water treated with 250 ppm 14% glutaraldehyde and 3% quaternary ammonium compound template with 1% *P. putida* genomic DNA and 1% *T. aromatica* genomic DNA
9	*P. puti* acrB/mexD_U and *P. puti* acrB_D primers against NTC
10	100 bp ladder
11	*T.aro* acrB2_U and *T.aro* acrB2/*T.aro* mexD_D primers against untreated surface water template with 1% *P. putida* genomic DNA and 1% *T. aromatica* genomic DNA
12	*T.aro* acrB2_U and *T.aro* acrB2/*T.aro* mexD_D primers against surface water treated with 250 parts per million (ppm) bronopol template with 1% *P. putida* genomic DNA and 1% *T. aromatica* genomic DNA
13	*T.aro* acrB2_U and *T.aro* acrB2/*T.aro* mexD_D primers against surface water treated with 250 ppm 15% glutaraldehyde template with 1% *P. putida* genomic DNA and 1% *T. aromatica* genomic DNA
14	*T.aro* acrB2_U and *T.aro* acrB2/*T.aro* mexD_D primers against surface water treated with 250 ppm DBNPA template with 1% *P. putida* genomic DNA and 1% *T. aromatica* genomic DNA
15	*T.aro* acrB2_U and *T.aro* acrB2/*T.aro* mexD_D primers against surface water treated with 250 ppm 14% glutaraldehyde and 3% quaternary ammonium compound template with 1% *P. putida* genomic DNA and 1% *T. aromatica* genomic DNA
16	*T.aro* acrB2_U and *T.aro* acrB2/*T.aro* mexD_D primers against NTC
17	100 bp ladder

Supplementary Figure 6. Gel electrophoresis in 1.5% agarose for 45 minutes at 100 volts and stained using Gel Red dye of PCR products using select MDREP primers targeting *mexD* from *P. putida* and *qacA1* from *P. putida* tested against DNA extracted from pure cultures and fresh water surface samples with and without biocide treatments, spiked with 1% DNA template from *P. putida* and *T. aromatica*.

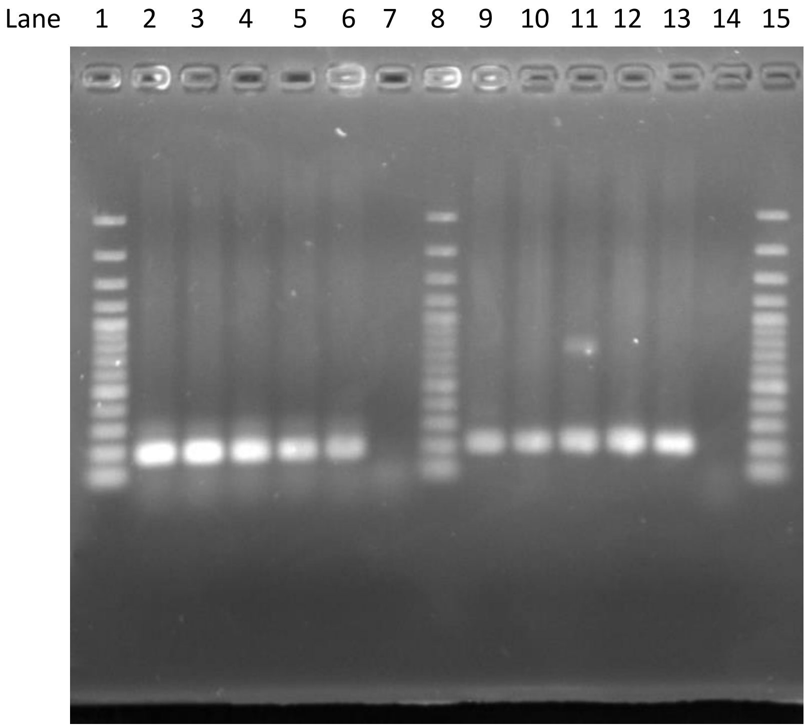

Lane	Sample
1	100 bp ladder
2	*P. puti* acrB/mexD_U and *T. aro* acrB2/*P. puti* mexD_D primers against untreated surface water template with 1% *P. putida* genomic DNA and 1% *T. aromatica* genomic DNA
3	*P. puti* acrB/mexD_U and *T. aro* acrB2/*P. puti* mexD_D primers against surface water treated with 250 parts per million (ppm) bronopol template with 1% *P. putida* genomic DNA and 1% *T. aromatica* genomic DNA
4	*P. puti* acrB/mexD_U and *T. aro* acrB2/*P. puti* mexD_D primers against surface water treated with 250 ppm 15% glutaraldehyde template with 1% *P. putida* genomic DNA and 1% *T. aromatica* genomic DNA
5	*P. puti* acrB/mexD_U and *T. aro* acrB2/*P. puti* mexD_D primers against surface water treated with 250 ppm DBNPA template with 1% *P. putida* genomic DNA and 1% *T. aromatica* genomic DNA
6	*P. puti* acrB/mexD_U and *T. aro* acrB2/*P. puti* mexD_D primers against surface water treated with 250 ppm 14% glutaraldehyde and 3% quaternary ammonium compound template with 1% *P. putida* genomic DNA and 1% *T. aromatica* genomic DNA
7	*P. puti* acrB/mexD_U and *T. aro* acrB2/*P. puti* mexD_D primers against NTC
8	100 bp ladder
9	*P. puti* qacA1/3_U and *P. puti* qacA1_D primers against untreated surface water template with 1% *P. putida* genomic DNA and 1% *T. aromatica* genomic DNA
10	*P. puti* qacA1/3_U and *P. puti* qacA1_D primers against surface water treated with 250 parts per million (ppm) bronopol template with 1% *P. putida* genomic DNA and 1% *T. aromatica* genomic DNA
11	*P. puti* qacA1/3_U and *P. puti* qacA1_D primers against surface water treated with 250 ppm 15% glutaraldehyde template with 1% *P. putida* genomic DNA and 1% *T. aromatica* genomic DNA
12	*P. puti* qacA1/3_U and *P. puti* qacA1_D primers against surface water treated with 250 ppm DBNPA template with 1% *P. putida* genomic DNA and 1% *T. aromatica* genomic DNA
13	*P. puti* qacA1/3_U and *P. puti* qacA1_D primers against surface water treated with 250 ppm 14% glutaraldehyde and 3% quaternary ammonium compound template with 1% *P. putida* genomic DNA and 1% *T. aromatica* genomic DNA
14	*P. puti* qacA1/3_U and *P. puti* qacA1_D primers against NTC
15	100 bp ladder
